# Nonsense-mediated decay influences position-dependent effects of *SCN2A* premature stop codons on neuronal excitability and behavior

**DOI:** 10.1038/s41380-026-03723-z

**Published:** 2026-07-06

**Authors:** Ahmad Al Saneh, Maria Fernanda Hermosillo Arrieta, Lionel Gissot, Madison O’Bryan, Rui Li, Gordon Buchanan, Christopher A. Ahern, Aislinn J. Williams

**Affiliations:** 1 Department of Molecular Physiology and Biophysics, University of Iowa, Iowa City, IA 52242 USA; 2 Iowa Neuroscience Institute, University of Iowa, Iowa City, IA 52242 USA; 3 Iowa: Discovering Research Experiences and Mentorship Postbaccalaureate Program, University of Iowa, Iowa City, IA 52242 USA; 4 Department of Neurology, University of Iowa, Iowa City, IA 52242 USA; 5 Department of Psychiatry, University of Iowa, Iowa City, IA 52242 USA

**Keywords:** Biochemistry, Genetics

## Abstract

*SCN2A* encodes the voltage-gated sodium channel Na_V_1.2, a central regulator of action potential initiation and propagation in glutamatergic neurons, and one of the strongest single-gene risk factors for autism spectrum disorder. Premature termination codons in *SCN2A* are widely considered to produce uniform haploinsufficiency through nonsense-mediated mRNA decay, an assumption that underpins current mechanistic and therapeutic models. We generated two mouse lines carrying patient-derived mutations – *Scn2a*^*Y84X/+*^ (p.Tyr84UAA; early coding sequence) and *Scn2a*^*R1627X/+*^ (p.Arg1627UGA; terminal coding exon). We assessed allele-specific mRNA expression, Na_V_1.2 protein expression, ex vivo whole-cell recordings, and behavioral phenotypes in these mice. Allele-specific RNA handling diverged by position: mRNA carrying Y84X engaged partial nonsense-mediated decay, whereas R1627X transcripts were at allelic balance. Despite this difference in RNA fate, Na_V_1.2 protein was comparably reduced in both lines. Both variants slowed the action potential upstroke, with a larger decrement in *Scn2a*^*Y84X/+*^. Spike threshold was depolarized only in *Scn2a*^*Y84X/+*^. Mutant neurons showed reduced firing near rheobase. Both lines exhibited increased grooming, but *Scn2a*^*Y84X/+*^ alone showed greater exploration and a male-predominant rotarod learning deficit. Locomotion, sociability, and sensorimotor gating were preserved. In maximal electroshock testing, mortality was reduced in both lines without changes in seizure threshold or severity. Our results show that *SCN2A* premature termination codon position determines allele-specific effects on neuronal excitability and behavior, where both NMD and phenotypes of the *Scn2a*^*Y84X/+*^ line are more penetrant. These data challenge the assumption of uniform haploinsufficiency and directly support allele-tailored mechanistic studies and therapeutic strategies.

## Introduction

Voltage-gated sodium channels (VGSCs) are transmembrane proteins that allow Na^+^ into excitable cells, including neurons [1, 2]. Na_V_1.2, whose alpha subunit is encoded by the gene *SCN2A*, is a VGSC involved in the generation, propagation, and backpropagation of action potentials in glutamatergic neurons [3–5]. *SCN2A*-related disorders manifest in a highly heterogeneous set of clinical diagnoses. Currently, *SCN2A* is the highest monogenetic risk gene for autism [4, 6, 7], and it has been linked to increased risk for many neurological and neurodevelopmental disorders, including epileptic encephalopathies (EE) [8–11], intellectual disability (ID) [12, 13], and ataxia [14]. Genetic variation in *SCN2A* has also been linked to bipolar disorder [15–17] and schizophrenia [18–20].

Mutations in *SCN2A* are generally classified as gain-of-function (GOF) or loss-of-function (LOF). GOF mutations increase sodium influx which promotes increased neuronal excitability and can underlie disorders such as early-onset epilepsy [7, 11, 21]. LOF variants reduce sodium current via impaired trafficking, channel activity, or protein truncation and are often associated with autism spectrum disorder (ASD) and ID, sometimes accompanied by seizures [4, 13]. Premature termination codons (PTCs) – non-native stop codons in the protein’s reading frame – are one type of LOF mutation. PTCs, also called “nonsense” mutations, are hypothesized to result in Na_V_1.2 haploinsufficiency, since one copy of the gene is unaffected and the PTC-bearing copy generates mRNA presumed to undergo nonsense-mediated decay (NMD) [22, 23]. However, NMD is influenced by many factors, including developmental stage, tissue-specific effects, and PTC location [24]. The effects of different *SCN2A* PTCs and the extent of NMD on neuronal and behavioral phenotypes remain unknown.

Existing *Scn2a* LOF mouse models demonstrate impairments in standard learning and memory assays, but results for other behavioral assays are variable [25]. Few patient-derived *Scn2a* mouse models exist [26–28], with only one modeling a nonsense mutation [29]. Not all LOF models show similar phenotypes, which highlights the importance of studying individual patient-derived mutations. Additionally, the question of whether all truncating variants yield similar outcomes remains unclear, especially since many are rare and de novo.

Here, we report the generation and characterization of two novel mouse models carrying the patient-derived PTC variants *Scn2a-*p.Tyr84X (Y84X) and -p.Arg1627X (R1627X). The Y84X variant results in an early truncation in the N-terminal cytoplasmic region, while the R1627X mutation occurs within the fourth transmembrane domain of the protein. We assessed the effects of each mutation on mRNA, Na_V_1.2 protein, neuronal excitability, and behavior. Surprisingly, NMD in the brains of *Scn2a* PTC mice was incomplete (Y84X) or absent (R1627X), suggesting that not all *Scn2a* PTCs are regulated identically by NMD in the brain. Further, both lines showed decreased total Na_V_1.2 protein but at levels statistically above 50%, as would be assumed for pure haploinsufficiency. Slice electrophysiology revealed allele-specific slowing of action potential upstroke compared to WT, as well as a threshold difference confined to *Scn2a*^*Y84X/+*^, consistent with allele-dependent impacts on spike-initiation dynamics.

Our behavioral assessment revealed a mix of distinct phenotypes across both lines, including increased exploration, diminished motor learning, and genotype-dependent decreased mortality in an induced seizure assay. Our data suggest that PTC mutations in Na_V_1.2 result in cellular and behavioral phenotypes that are both overlapping and mutation-specific, challenging the assumption that all PTCs would lead to similar phenotypes regardless of their genomic location.

## Materials and methods

### Animals

Transgenic mice were generated in collaboration with the Iowa Genome Editing Core using CRISPR/Cas12 (*Scn2a*^*Y84X/+*^) and CRISPR/Cas9 (*Scn2a*^*R1627X/+*^). Both lines were generated on the C57BL/6 J strain from Jackson Labs (Strain #000664, Bar Harbor, ME). Transgenic mouse generation and validation details can be found in the Supplemental Information. All experiments were conducted according to the National Institutes of Health guidelines for animal care and were approved by the Institutional Animal Care and Use Committee at the University of Iowa. Sample sizes were determined based on prior studies, anticipated availability of age-matched animals from the breeding colony, and the goal of obtaining sufficient biological replication across genotypes and sexes. Final sample sizes are reported for each assay, and analyses incorporated sex as a biological variable where appropriate.

### Biochemistry

#### Nonsense-Mediated decay

Whole brains were harvested from adult mice (*n* = 3 per genotype; *Scn2a*^+/+^, *Scn2a*^*Y84X/+*^, *Scn2a*^*R1627X/+*^), flash-frozen, and homogenized in TRIzol reagent (Thermo Fisher Scientific). Total RNA was isolated according to the manufacturer’s protocol. Primer sequences and PCR conditions are provided in the Supplemental Information.

#### Na_V_1.2 protein expression levels

Brains were collected from P30 mice following isoflurane anesthesia and decapitation (*Scn2a*^*+/+*^, *n* = 8; *Scn2a*^*Y84X/+*^, *n* = 7; *Scn2a*^*R1627X/+*^, n = 8). One hemisphere was homogenized in RIPA buffer with protease inhibitors, and total protein concentration was measured by Bradford assay. Protein lysates (0.026 mg/ml) were analyzed on the Jess™ Simple Western™ capillary immunoassay system (ProteinSimple) using a C-terminal monoclonal anti-SCN2A antibody (5H10.2, Millipore Sigma) or an N-terminal polyclonal anti-SCN2A antibody (ASC-002, Alomone Labs) after normalization using the total protein assay (DM-TP01, ProteinSimple).

### Ex Vivo electrophysiology

On the day of acquisition, the order of experimentation (mouse line and genotype) was determined by coin flip to randomize. Acute coronal brain slices (250 µm) containing medial prefrontal cortex were prepared from P25-35 mice, including 11 *Scn2a*^*+/+*^ (WT) mice, 6 *Scn2a*^*Y84X/+*^ mice, and 6 *Scn2a*^*R1627X/+*^ mice. Recordings were obtained from layer 5b thick-tufted pyramidal tract (PT) neurons positioned ~1.5 mm ventral to the dorsal pia and ~450 µm from the midline in coronal sections, as previously described [30].

Whole-cell recordings were made with MultiClamp 700B amplifiers (Molecular Devices) controlled by Clampex and analyzed in Clampfit. Solution recipes and recording criteria are provided in the Supplemental Information.

### General behavioral procedures

All mice were adults (16–30 weeks) at the time of behavioral testing. Mice were housed under regular light cycle with lights on/off at 0900/2100 DST (0800/2000 non-DST). All experiments were conducted during the animals’ light cycle. Mice were same-sex housed in mixed-genotype groups (2–5 mice per cage) on vented cage racks with nesting material as enrichment. Mice were provided with food and water *ad libitum*. Mice were acclimatized for 30 min in the room where assays were conducted before initiating each test. Equipment was cleaned between trials with 70% ethanol, and the experimenters wore appropriate protective personal equipment when handling the mice. Mice were randomized and color-coded with Sharpie markers on their tails at the beginning of behavioral testing to maintain blinding to experimental groups during testing and scoring. Additional details are provided in the Supplemental Information.

#### Elevated zero maze

Each mouse was tested on an elevated zero maze for 5 min under ~245 lux illumination. Distance traveled and time spent in open versus closed sections were quantified using EthoVision XT 17 (Noldus). Mice were excluded from statistical analysis if they could not complete the trial (i.e. jumped off the apparatus). Three *Scn2a*^*Y84X/+*^ and one *Scn2a*^*R1627X/+*^ mice were excluded from this task for this reason. Y84X *n* = 33 (WTs: *n* = 6 males, *n* = 9 females; *Scn2a*^*Y84X/+*^: *n* = 11 males, *n* = 7 females). R1627X *n* = 41 (WTs: *n* = 8 males, *n* = 13 females; *Scn2a*^*R1627X/+*^: *n* = 10 males, *n* = 10 females).

#### Open field test

Mice were tested in a black plastic open field arena for 10 min under ~115 lux illumination. Distance traveled and thigmotaxis were quantified using EthoVision XT 17. Grooming behavior was scored offline by a blinded observer using BORIS software [31]. Y84X *n* = 36 (WTs: *n* = 6 males, *n* = 9 females; *Scn2a*^*Y84X/+*^: *n* = 12 males, *n* = 9 females). R1627X *n* = 42 (WTs: *n* = 8 males, *n* = 13 females; *Scn2a*^*R1627X/+*^: *n* = 10 males, *n* = 11 females).

#### Rotarod

Motor coordination was assessed on an accelerating rotarod (4–40 rpm over 5 min). Mice underwent three trials per day for five consecutive days. Latency to fall or passive rotation was recorded. Y84X *n* = 36 (WTs: *n* = 6 males, *n* = 9 females; *Scn2a*^*Y84X/+*^: *n* = 12 males, *n* = 9 females). R1627X *n* = 42 (WTs: *n* = 8 males, *n* = 13 females; *Scn2a*^*R1627X/+*^: *n* = 10 males, *n* = 11 females).

#### Three-chamber social task

Mice were tested in a three-chamber apparatus for sociability following a 10-min habituation. A novel mouse and a novel object were placed in opposite chambers, and mice were allowed to explore for 10 min. Distance traveled, time spent per chamber, and interaction frequency were measured using EthoVision XT 17. Mice were excluded from statistical analysis if they could not complete a phase (i.e. jumped out of the testing arena). Two male *Scn2a*^*Y84X/+*^ mice were excluded from the sociability phase for this reason. Y84X *n* = 35 & 37 (WTs: *n* = 11 males, *n* = 10 females; *Scn2a*^*Y84X/+*^: *n* = 6 & 8 males, *n* = 8 females). R1627X *n* = 27 (WTs: *n* = 8 males, *n* = 7 females; *Scn2a*^*R1627X/+*^: *n* = 6 males, *n* = 6 females).

#### Prepulse inhibition

Acoustic startle and prepulse inhibition were assessed using the SR-LAB system (San Diego Instruments). Mice were tested using randomized pulse-alone and prepulse trials (70–80 dB) preceding a 120 dB startle. Percent PPI was calculated relative to pulse-alone responses. Y84X *n* = 37 (WTs: *n* = 11 males, *n* = 10 females; *Scn2a*^*Y84X/+*^: *n* = 8 males, *n* = 8 females). R1627X *n* = 27 (WTs: *n* = 8 males, *n* = 7 females; *Scn2a*^*R1627X/+*^: *n* = 6 males, *n* = 6 females).

#### EEG and EMG headmount implantation and monitoring

Procedures followed published methods [32]. Signals were recorded continuously, digitized at 1000 Hz, and analyzed using Sirenia Seizure Pro software. Automated seizure detection was followed by manual verification.

#### Maximal electroshock (MES) seizure induction

MES seizures were induced as previously described [32] using saline-moistened ear clips and incrementally increasing current until generalized tonic–clonic seizures occurred. Outcomes included seizure threshold, mortality, seizure-free proportion, and extension-to-flexion ratio. Y84X *n* = 26 (WTs: *n* = 8 males, *n* = 6 females; *Scn2a*^*Y84X/+*^: *n* = 6 males, *n* = 6 females). R1627X *n* = 24 (WTs: *n* = 6 males, *n* = 5 females; *Scn2a*^*R1627X/+*^: *n* = 6 males, *n* = 7 females).

### Statistics

Statistical analyses were performed using OriginPro (OriginLab, Northampton, MA), GraphPad Prism 10.5 (GraphPad Software, San Diego, CA), and Rstudio (R4.4.0, afex 1.4-1, emmeans 1.11.1, lme4 1.1-37, lmerTest 3.1-3, rcompanion 2.5.0, survival 3.8-3). Data were analyzed using the statistical tests specified in the Results, figure legends, and supplementary tables (two-sided two-way ANOVA, ranked two-way ANOVA, Fisher’s exact test, Chi-square test of independence, log-rank test, linear mixed effects model, or linear model with appropriate follow-up testing). Results are presented as mean ± SEM or SD as indicated. Behavioral data were analyzed with sex included as a biological variable. Normality tests were run to determine the appropriate statistical method for analysis of each experiment. Outlier tests were run using ROUT with a Q = 1%. Statistical significance was set at *p* < 0.05.

## Results

### NMD in *Scn2a* PTC mouse models

**Fig. 1:**
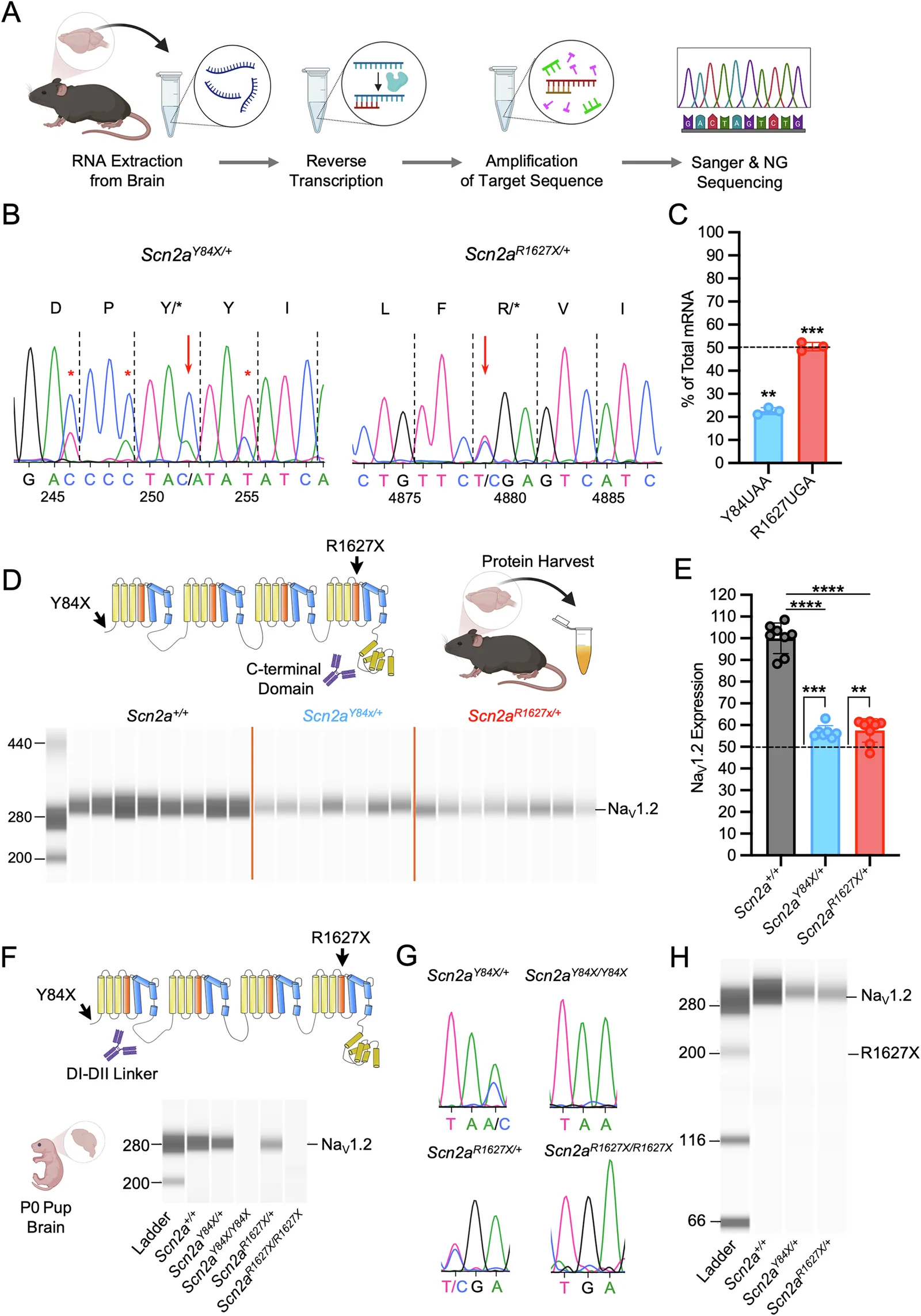
Biochemical characterization of *Scn2a* mouse models. **A** Schematic representation of experimental design performed to quantify NMD in *Scn2a* mouse models. **B** Sanger sequencing of *Scn2a* mRNA shows the Y84UAA mutation at 252 bp (Left) and R1626UGA at 4879 bp (Right). Red asterisks indicate silent mutations introduced via CRISPR during the generation of *Scn2a*^*Y84X/+*^ mice. **C** NGS quantifies transcripts containing PTCs in *Scn2a*^*Y84X/+*^ and *Scn2a*^*R1627X/+*^. Male and female mice are represented by circle and diamond symbols, respectively (*n* = 3 mice per genotype, *p* < 0.001). **D Top**, Monoclonal anti-Na_V_1.2 antibody binds to the C-terminal domain. Arrows: two PTCs corresponding to each *Scn2a* mouse model. **Bottom**, Representative Jess automated capillary immunoassay, rendered as a “western blot” showing expression of Na_V_1.2 in half-brain lysates. **E** Bar graph quantification of peak intensity shown in (**D**) displaying decreased Na_V_1.2 protein in mutant brains compared to WT littermates (ANOVA, *p* < 0.0001) but over 50% (One-sample t-test, *p* < 0.005). Na_V_1.2 protein levels were normalized against total protein for each sample, then compared to WT mean value. Male and female mice are represented by circle and diamond symbols, respectively (WT: 2 males, 6 females; *Scn2a*^*Y84X/+*^: 4 males, 3 females; *Scn2a*^R1627X/+^: 4 males, 4 females). Data are mean ± SD. **F Top**, Polyclonal anti-Na_V_1.2 antibody binds to the DI-DII linker. **Bottom**, anti-Na_V_1.2 western blot shows no reactivity in *Scn2a*^*Y84X/Y84X*^ and *Scn2a*^*R1627X/R1627X*^ neonatal brain. **G** Sanger sequencing data validate PTCs in both alleles of *Scn2a*^*Y84X/Y84X*^ and *Scn2a*^*R1627X/R1627X*^ neonatal mouse brains. **H** Na_V_1.2 western blot using DI-DII linker-targeting antibody shows absence of a truncated Na_V_1.2 species in P30 *Scn2a*^*R1627X/+*^ brain.

Total RNA was isolated from whole brains of adult *Scn2a* mice for sequence confirmation and quantification (Fig.1A). Chromatograms from *Scn2a*^*Y84X/+*^ mouse brains showed mixed bases at the Y84 locus, where nucleotide 252 (C/A) yielded TAC (Tyr) in one allele and TAA (PTC) in the other (Fig.1B). Similarly, at the R1627 locus from *Scn2a*^*R1627X/+ *^mice, chromatograms showed mixed bases at nucleotide 4879 (C/T), corresponding to CGA (Arg) and TGA (PTC), respectively. These data confirm the presence of the expected heterozygous PTCs in both mouse models (Fig.1B).

Allele fractions were then quantified by targeted next-generation sequencing (NGS) (Fig.1C). The mutant-allele fraction averaged 22.5 ± 0.8% in *Scn2a*^*Y84X/+*^ brains and 50.49 ± 1.0% in *Scn2a*^*R1627X/+*^ brains (*p* < 0.001), confirming expression of the PTC-bearing transcripts in both genotypes. Relative to the predicted allelic balance (50%), the Y84X mutant allele was under-represented (22.5%, *p* < 0.001), consistent with incomplete NMD at this site. By contrast, R1627X-bearing transcripts were at balance (50.49%, *p* > 0.6), suggesting no detectable NMD occurred to this PTC-bearing transcript. Transcript abundances showed no apparent sex-dependent differences; however, group sizes were optimized for primary NMD quantification and were insufficient to power a formal genotype-by-sex interaction analysis (Supplemental Information).

### Na_V_1.2 expression in *Scn2a* PTC mice

Na_V_1.2 abundance was quantified in half-brain lysates from P30 *Scn2a* mice (Fig. 1D). Across WT (*n* = 8), *Scn2a*^*Y84X/+*^ (*n* = 7), and *Scn2a*^*R1627X/+*^ (*n* = 8) cohorts, Na_V_1.2 levels differed significantly (*p* < 0.0001). Relative to WT, Na_V_1.2 was reduced to 56.8 ± 2.9% in *Scn2a*^*Y84X/+*^ and 57.5 ± 5.4% in *Scn2a*^*R1627X/+*^ (mean ± SD) (Fig. 1E). Despite this reduction, Na_V_1.2 remained > 50% of WT in each mutant line (*p* < 0.005 for both), with no significant difference detected between the two variants. Additional analysis revealed no significant effect of sex and no significant genotype-by-sex interaction on Na_V_1.2 protein abundance in *Scn2a*^*Y84X/+*^ and *Scn2a*^*R1627X/+*^ heterozygous mice (sex effect: *p* = 0.214; interaction: *p* = 0.171. Supplemental Information).

To further assess whether either PTC allele produces detectable protein, we examined brain lysates from P0–P1 *Scn2a*^*Y84X/Y84X*^ and *Scn2a*^*R1627X/R1627X*^ mice using an antibody directed against the DI–DII intracellular linker (Fig. 1F, G). Under these conditions, Na_V_1.2 immunoreactivity was not detected in either homozygous genotype, consistent with loss of full-length protein expression. In addition, we found no evidence of a truncated Na_V_1.2 species in P30 *Scn2a*^*R1627X/+*^ brain lysates, indicating that the R1627X variant does not produce a stable truncated protein in vivo (Fig. 1H).

To determine whether the above-50% Na_V_1.2 levels in each of the two *Scn2a* mouse model brains could reflect translational readthrough at either PTC, we resolved P30 brain lysates from WT (*n* = 3), *Scn2a*^*Y84X/+*^ (*n* = 3), and *Scn2a*^*R1627X*/+^ (*n* = 3) mice by SDS-PAGE, excised the full-length Na_V_1.2 band from each lane, and analyzed the extracted protein by mass spectrometry (Supplementary Fig. 1). Peptide coverage at codon 84 in *Scn2a*^*Y84X*/+^ samples detected only tyrosine (the wild-type residue), with no evidence of amino acid substitution or misincorporation. Similarly, peptide coverage at codon 1627 in *Scn2a*^*R1627X*/+^ samples detected only arginine. These data indicate that the Na_V_1.2 protein present in both mutant lines derives entirely from the wild-type allele and that translational readthrough does not contribute detectably to the full-length protein pool. The mechanism underlying the above-50% Na_V_1.2 levels therefore remains unresolved and may reflect compensatory upregulation of WT allele translation or reduced protein turnover.

### Electrophysiological assessment of *Scn2a* PTC mouse models

**Fig. 2:**
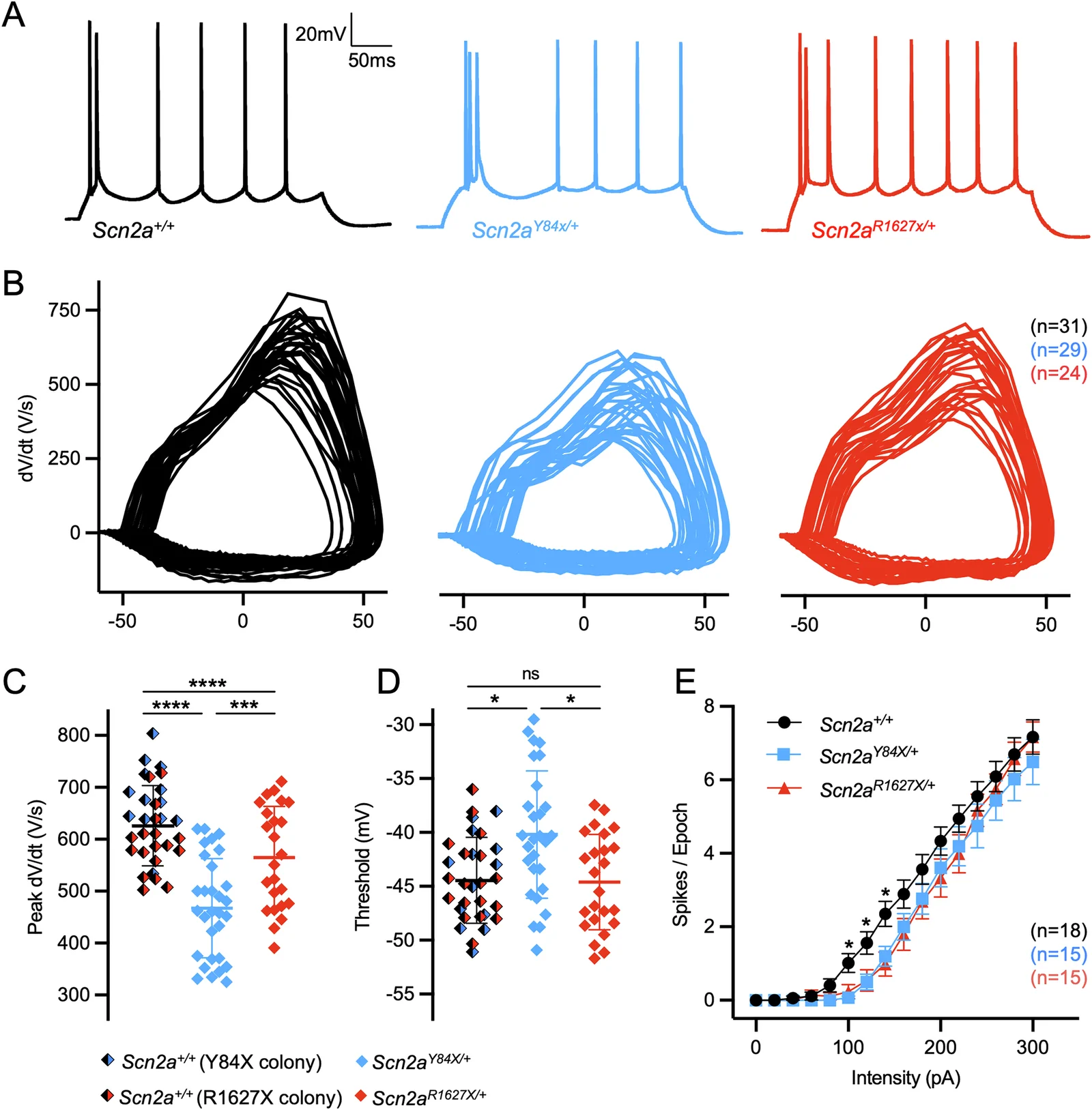
Electrophysiological characterization of *Scn2a* mouse models. **A** Action potentials generated by current injection (300pA, 300 ms) in *Scn2a*^+/+^ (black), *Scn2a*^*Y84X/+*^ (cyan) and *Scn2a*^*R1627X/+*^ (red) cortical pyramidal neurons. **B** Phase-plane plots of all action potentials recorded (same color-coding as (**A**)). **C** Maximum speed of the rising phase of the APs. **D** Threshold of APs . Data are mean ± SD. One-way ANOVA, followed by Tukey’s post hoc test for multiple comparisons. **E** F–I curves of cortical pyramidal neurons from WT (*n* = 18), *Scn2a*^*Y84X/+*^ (*n* = 15), and *Scn2a*^*R1627X/+*^ (*n* = 15) mice showing mean spikes ± SEM. Group differences were assessed per step by one-way ANOVA with Tukey’s HSD post hoc tests.

Acute medial prefrontal cortex (mPFC) slices from *Scn2a* mice (ages P25-P30) were examined by performing whole-cell current clamp on layer 5b pyramidal tract (PT) neurons [5, 30, 33] (see Supplemental Information). Action potentials (APs) were evoked with a 300 pA, 300 ms depolarizing step (Fig. 2A). Phase-plane plots of dV/dt versus membrane voltage were then used to quantify the kinetics of the rising phase of the first AP spike (Fig. 2B). This approach differentiates contributions of axonal initial segment (AIS) and somatodendritic sodium channels [5].

Somatic peak dV/dt differed markedly across genotypes. Neurons from WT mice exhibited a rapid upstroke, with a mean of 630.3 ± 78.47 V s⁻¹ (mean ± SD, *n* = 31 cells) (Fig. 2C), while neurons expressing *Scn2a*^*R1627X/+*^ exhibited slower kinetics (564.9 ± 98.3 V s⁻¹, *n* = 24; *p* < 0.05). APs recorded from *Scn2a*^*Y84X/+*^ neurons were significantly slower than both WT (*p* < 0.0001) and *Scn2a*^*R1627X/+*^ (*p* < 0.001) (462.1 ± 92.7 V s⁻¹, *n* = 29). Although these reductions are consistent with previous observations that Na_V_1.2 heterozygous LOF mutations attenuate the velocity of membrane depolarization [34, 35], the more pronounced reduction in *Scn2a*^*Y84X/+*^ neurons suggests that the Y84X mutation results in a more severe functional deficit than R1627X.

AP spike thresholds were similar in WT neurons (−44.2 ± 3.9 mV) and *Scn2a*^*R1627X/+*^ neurons (−44.5 ± 4.4 mV; *p* > 0.05) (Fig. 2D). In contrast, *Scn2a*^*Y84X/+*^ neurons required greater depolarization to spike (−40.7 ± 6.3 mV, *p* < 0.05 vs wild type and *Scn2a*^*R1627X/+*^), indicating that this PTC allele shifts the AP threshold to a more depolarized potential (Fig. 2D).

To evaluate how PTC alleles affect neuronal excitability, we injected incrementing 300 ms current steps (F–I curves, Fig. 2E). Both PTC models fired fewer spikes than WT at near-rheobase inputs (100–140 pA). At higher current injections however, firing in *Scn2a* PTC neurons converged toward the WT phenotype.

### Weight

*Scn2a*^*Y84X/+*^ and *Scn2a*^*R1627X/+*^ mice were viable and born at expected Mendelian ratios. Across both lines, *Scn2a* PTC mice weighed slightly less than WT littermates (Y84X *n* = 37, *p* < 0.01; R1627X *n* = 33, *p* < 0.01). *Scn2a*^*Y84X/+*^ mice were on average 2.03 g smaller than WT (Figure S2A), while *Scn2a*^*R1627X/+*^ mice were 2.59 g smaller on average (Figure S2B). We observed a main effect of sex, with females weighing less than males in both lines (Supplementary Table 1). Overall, we would not expect these weight differences to impede the animals’ ability to perform behavioral tasks.

### Exploratory and locomotor behavior

**Fig. 3:**
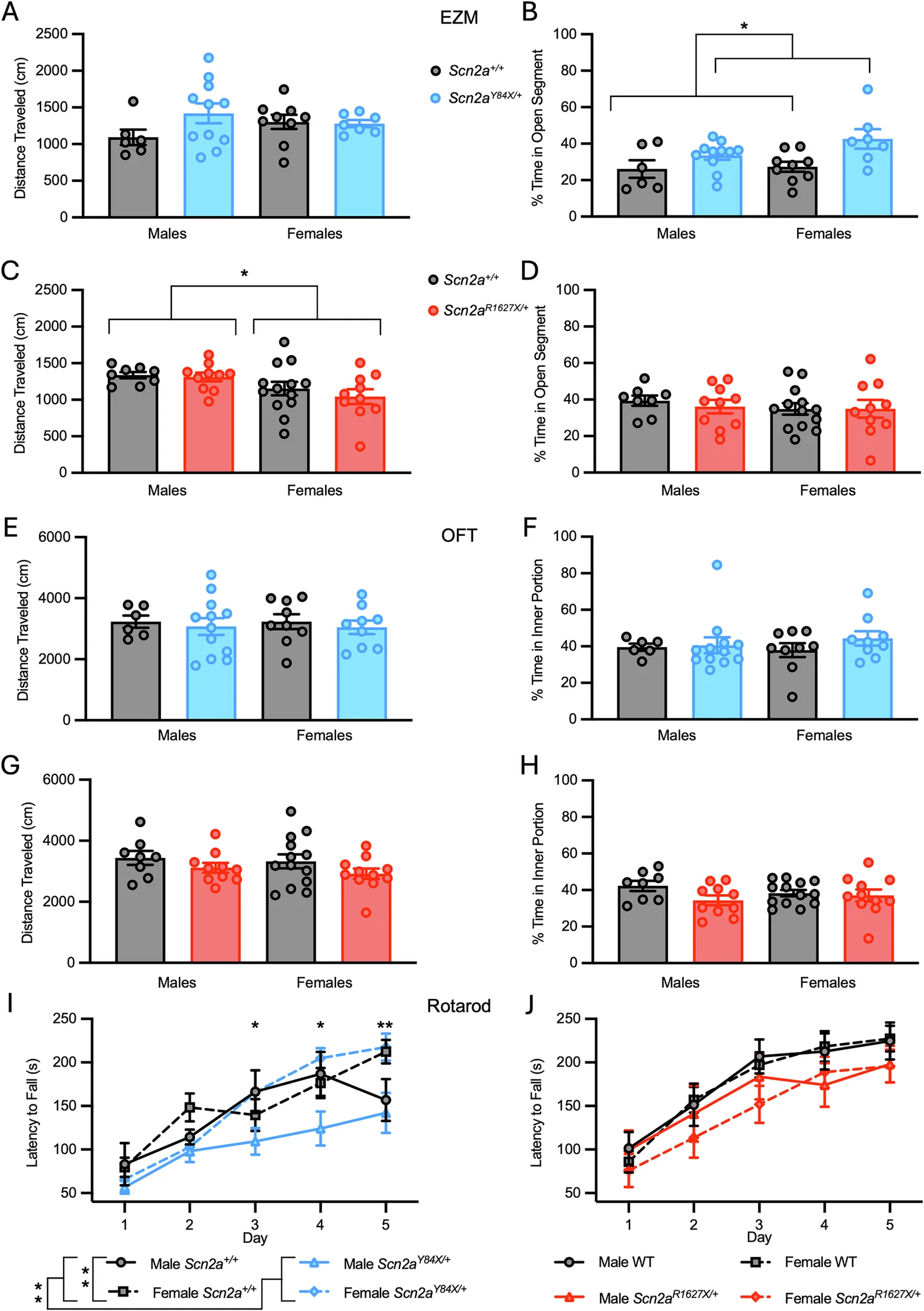
*Scn2a*^*Y84X/+*^ mice exhibit diminished anxiety-like behaviors and alterations in motor behavior. **A** There were no differences in distance traveled in the Y84X line for the elevated zero maze (two-way ANOVA, genotype *p* = 0.204, sex *p* = 0.765, genotype*sex *p* = 0.144). **B** *Scn2a*^*Y84X/+*^ mice spent significantly more time in the open segment of the elevated zero maze compared to WT mice (two-way ANOVA, genotype *p* < 0.01, sex *p* = 0.177, genotype*sex *p* = 0.296). **C** Male mice in the R1627X line traveled longer distances in the elevated zero maze (two-way ANOVA, genotype *p* = 0.429, sex *p* = 0.012, genotype*sex *p* = 0.628). **D** There were no differences in time spent in the open segment of the elevated zero maze in the R1627X line (two-way ANOVA, genotype *p* = 0.678, sex *p* = 0.458, genotype*sex *p* = 0.668). **E** There were no differences in distance traveled (two-way ANOVA, genotype *p* = 0.519, sex *p* = 0.958, genotype*sex *p* = 0.961) or (**F**) time spent in the inner portion of the arena of the open field task in the Y84X line (two-way ANOVA, genotype *p* = 0.987, sex *p* = 0.238, genotype*sex *p* = 0.394). **G** There were no differences observed in distance traveled (two-way ANOVA, genotype *p* = 0.085, sex *p* = 0.459, genotype*sex *p* = 0.846) or (**H**) time spent in the inner portion of the arena of the open field task in the R1627X line (two-way ANOVA, genotype *p* = 0.091, sex *p* = 0.805, genotype*sex *p* = 0.221). **I** We observed a genotype × session × sex interaction (linear-mixed effects model, *p* < 0.01) in the rotarod assay for the Y84X line. Post-hoc testing showed a difference between male *Scn2a*^*Y84X/+*^ and WT mice on sessions 3 and 4. Further, we observed a sex × session interaction effect (linear-mixed effects model, *p* < 0.01) between males and females on session 5, as well as a main effect of session (linear-mixed effects model, genotype *p* = 0.234, sex *p* = 0.067, session *p* < 0.01, genotype*sex *p* = 0.230, genotype*session *p* = 0.638). **J** No differences in rotarod performance were detected between mice in the R1627X line. All genotypes improved over the course of training (linear-mixed effects model, genotype *p* = 0.139, sex *p* = 0.640, session *p* < 0.01, genoty*p*e*sex *p* = 0.733, genotype*session *p* = 0.347, sex*session *p* = 0.485, genotype*sex*session *p* = 0.789). **A-J** Data are expressed as mean ± SEM. **A-B** *n* = 33 (WTs: *n* = 6 males, *n* = 9 females; *Scn2a*^*Y84X/+*^: *n* = 11 males, *n* = 7 females). **C-D** *n* = 41 (WTs: *n* = 8 males, *n* = 13 females; *Scn2a*^*R1627X/+*^: *n* = 10 males, *n* = 10 females). **E, F, I** *n* = 36 (WTs: *n* = 6 males, *n* = 9 females; *Scn2a*^*Y84X/+*^: *n* = 12 males, *n* = 9 females). **G, H, J** *n* = 42 (WTs: *n* = 8 males, *n* = 13 females; *Scn2a*^*R1627X/+*^: *n* = 10 males, *n* = 11 females).

We examined exploration and locomotion in *Scn2a* PTC mice. We observed no differences in distance traveled by *Scn2a*^*Y84X/+*^ mice (Fig. 3A, Supplementary Table 2) in the elevated zero maze. They spent more time exploring the open portions of the maze than WT littermates (Fig. 3B, Supplementary Table 3), with no main effect of sex or interaction effects. This may be evidence of reduced anxiety-like behavior or increased exploratory behavior in *Scn2a*^*Y84X/+*^ mice. In contrast, *Scn2a*^*R1627X/+*^ mice showed no differences in distance traveled or open segment exploration compared to their WT littermates (Fig. 3C-D, Supplementary Tables 2–3). We observed a main effect of sex on elevated zero maze distance traveled in the R1627X line, such that males explored slightly more than females (Supplementary Table 2). In the open field test, we observed no differences in distance traveled or time in the inner portion of the open field for either *Scn2a*^*Y84X/+*^ mice (Fig. 3E-F, Supplementary Tables 2–3) or *Scn2a*^*R1627X/+*^ mice (Fig. 3G-H, Supplementary Tables 2-3). These findings indicate preserved baseline locomotion and increased willingness to explore high-risk areas of the elevated zero maze specifically in *Scn2a*^*Y84X/+*^ mice.

### Motor performance learning

We then used the accelerating rotarod test to investigate motor learning in *Scn2a* PTC mice, given the presence of Na_V_1.2 in cerebellar granule neurons and reports of movement disorders in patients with *SCN2A-*associated syndromes [4, 36]. *Scn2a*^*Y84X/+*^ mice displayed a significant genotype × session × sex interaction (Fig. 3I, Supplementary Table 4); post hoc testing revealed impaired motor learning selectively in male *Scn2a*^*Y84X/+*^ mice in sessions 3–4, with an additional sex difference in session 5. In contrast, *Scn2a*^*R1627X/+*^ mice showed normal rotarod learning with a main effect of session and no genotype or sex effects (Fig. 3J, Supplementary Table 4). Therefore, the Y84X mutation is associated with a male-predominant impairment in motor performance learning on the accelerating rotarod task, while the R1627X mutation does not impact rotarod performance.

### Grooming behavior

**Fig. 4:**
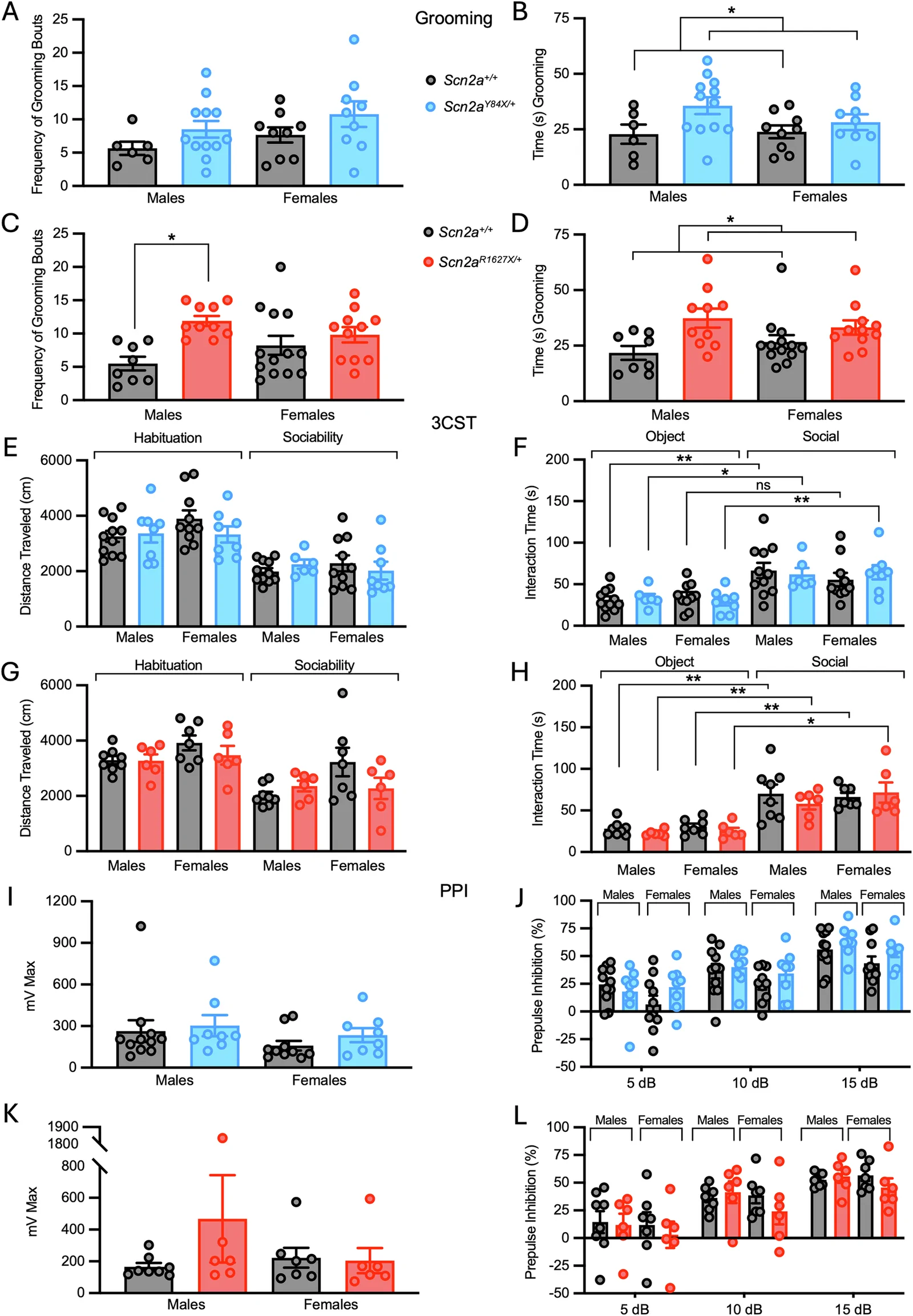
*Scn2a*^*Y84X/+*^ and *Scn2a*^*R1627X/+*^ mice exhibit restrictive and repetitive behaviors. **A** There were no differences in grooming frequency in the Y84X line for the open field test (two-way ANOVA, genotype *p* = 0.054, sex *p* = 0.159, genotype*sex *p* = 0.926). **B** *Scn2a*^*Y84X/+*^ mice spent significantly more time grooming compared to WT mice (two-way ANOVA, genotype *p* = 0.031, sex *p* = 0.408, genotype*sex *p* = 0.273). **C** *Scn2a*^*R1627X/+*^ male mice had a higher number of grooming bouts compared to WT males (ranked two-way ANOVA, genotype *p* < 0.01, sex *p* = 0.864, genotype*sex *p* = 0.047). **D** *Scn2a*^*R1627X/+*^ mice spent more time grooming in the open field assay (ranked two-way ANOVA, genotype *p* < 0.01, sex *p* = 0.865, genotype*sex *p* = 0.493). **E** There were no differences in distance traveled in the three-chamber social task (linear-mixed effects model, genotype *p* = 0.718, sex *p* = 0.579, *p*hase *p* < 0.01, genotype*sex *p* = 0.198, genotype*phase *p* = 0.167, sex*phase *p* = 0.112, genoty*p*e*sex*phase *p* = 0.915). **F** WT females in the Y84X line did not show preference for the novel mouse in the sociability phase of the three-chamber social task (paired t-test, male WT *p* < 0.01, male *Scn2a*^*Y84X/+*^ *p* = 0.014, female WT *p* = 0.095, female *Scn2a*^*Y84X/+*^ *p* < 0.01; linear-mixed effects model, genotype *p* = 0.999, sex *p* = 0.701, cylinder *p* < 0.01, genotype*sex *p* = 0.835, genoty*p*e*cylinder *p* = 0.664, sex*cylinder *p* = 0.649, genotype*sex*cylinder *p* = 0.234). **G** There were no differences observed in distance traveled (linear-mixed effects model, genotype *p* = 0.334, sex *p* = 0.088, phase *p* < 0.01, genotype*sex *p* = 0.128, genotype*phase *p* = 0.760, sex*phase *p* = 0.536, genotype*sex*phase *p* = 0.081) or (**H**) time spent with the conspecific mice in the R1627X line in the three-chamber social task (paired t-test, male WT *p* < 0.01, male *Scn2a*^*R1627X/+*^ *p* < 0.01, female WT *p* < 0.01, female *Scn2a*^*R1627X/+*^ *p* = 0.026; linear-mixed effects model, genotype *p* = 0.388, sex *p* = 0.511, cylinder *p* < 0.01, genotype*sex *p* = 0.378, genotype*cylinder *p* = 0.863, sex*cylinder *p* = 0.901, genotype*sex*cylinder *p* = 0.396). **I** There were no differences in startle response (linear-mixed effects model, genotype *p* = 0.456, sex *p* = 0.179, block *p* = 0.015, genotype*sex *p* = 0.857, genotype*block *p* = 0.844, sex*block *p* = 0.827, genotype*sex*block *p* = 0.477) or (**J**) percent of prepulse inhibition in the Y84X line (linear-mixed effects model, genotype *p* = 0.203, sex *p* = 0.101, decibel *p* < 0.01, genotype*sex *p* = 0.325, genotype*decibel *p* = 0.745, sex*decibel *p* = 0.828, genotype*sex*decibel *p* = 0.249). **K** There were no differences in startle response (linear-mixed effects model, genotype *p* = 0.231, sex *p* = 0.312, block *p* = 0.058, genotype*sex *p* = 0.177, genotype*block *p* = 0.755, sex*block *p* = 0.583, genotype*sex*block *p* = 0.208) or (**L**) percent of prepulse inhibition in the R1627X line (linear-mixed effects model, genotype *p* = 0.523, sex *p* = 0.461, decibel *p* < 0.01, genotype*sex *p* = 0.370, genotype*decibel *p* = 0.971, sex*decibel *p* = 0.829, genotype*sex*decibel *p* = 0.662). **A-L** Data are expressed as mean ± SEM. **A,B** *n* = 36 (WTs: *n* = 6 males, *n* = 9 females; *Scn2a*^*Y84X/+*^: *n* = 12 males, *n* = 9 females). **C,D** *n* = 42 (WTs: *n* = 8 males, *n* = 13 females; *Scn2a*^*R1627X/+*^: *n* = 10 males, *n* = 11 females). **E,F** *n* = 35 & 37 (WTs: *n* = 11 males, *n* = 10 females; *Scn2a*^*Y84X/+*^: *n* = 6 & 8 males, *n* = 8 females). **G,H, K,L** *n* = 27 (WTs: *n* = 8 males, *n* = 7 females; *Scn2a*^*R1627X/+*^: *n* = 6 males, *n* = 6 females). **I-J** *n* = 37 (WTs: *n* = 11 males, *n* = 10 females**;** *Scn2a*^*Y84X/+*^: *n* = 8 males, *n* = 8 females).

We next assessed grooming behavior in the open field arena as a proxy for restrictive and repetitive behavior, a cardinal feature of autism [37]. *Scn2a*^*Y84X/+*^ mice showed no differences in grooming frequency but spent significantly more time grooming than their WT littermates (Fig. 4A-B), indicating prolonged grooming bouts. In the R1627X line, we observed a genotype × sex interaction in grooming frequency (Fig. 4C). Post-hoc analysis showed no difference between females; however, male *Scn2a*^*R1627X/+*^ mice groomed more frequently than their WT littermates. *Scn2a*^*R1627X/+*^ mice also spent more time grooming overall than WT littermates (Fig. 4D, Supplementary Table 5). Together, these findings suggest that both *Scn2a* PTC lines exhibit restrictive and repetitive grooming behavior.

### Social preference and sensorimotor gating

Social preference was assessed using the three-chamber task given the association between *SCN2A* and autism. *Scn2a*^*Y84X/+*^ mice showed comparable locomotor activity to WT littermates across phases, with higher exploration during habituation than sociability and no genotype or sex effects (Fig. 4E, Supplementary Table 6A-B). *Scn2a*^*Y84X/+*^ mice displayed a preference for interacting with novel mice compared to novel objects, similar to WT littermates (Fig. 4F). We observed similar locomotion and social preference behaviors in *Scn2a*^*R1627X/+*^ mice (Fig. 4G-H, Supplementary Table 6A-B). Overall, *Scn2a*^*Y84X/+*^ and *Scn2a*^*R1627X/+*^ mice display social preference similar to WT mice.

We used the prepulse inhibition task to measure sensorimotor gating, as impairments have been reported in autistic people [38, 39]. *Scn2a*^*Y84X/+*^ mice showed auditory startle responses similar to their WT littermates (Fig. 4I), as well as normal sensorimotor gating, with no significant effects of genotype or sex (Fig. 4J, Supplementary Table 7). We observed similar results for *Scn2a*^*R1627X/+*^ mice (Fig. 4K-L, Supplementary Table 7). Higher decibel prepulses yielded a higher percentage of prepulse inhibition across all mice, and mild startle habituation across blocks was only observed in the Y84X line without effects of genotype or sex (Figure S4 A-B). We conclude that *Scn2a*^*Y84X/+*^ and *Scn2a*^*R1627X/+*^ mice have adequate startle response and normal sensorimotor gating.

### Spontaneous seizures and MES threshold

**Fig. 5:**
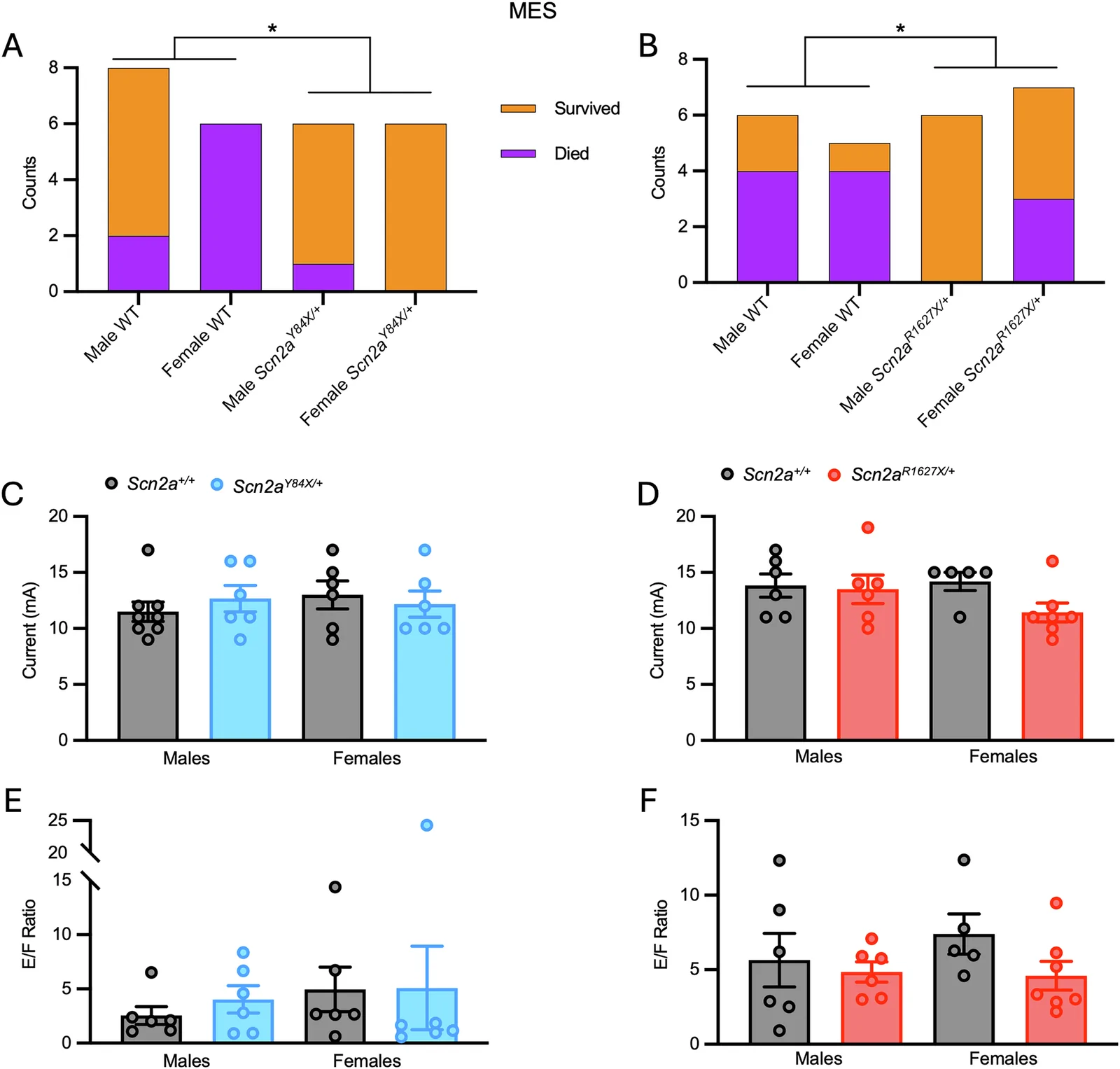
*Scn2a*^*Y84X/+*^ mice show decreased mortality in seizure induction assay. **A** No female *Scn2a*^*Y84X/+*^ mice died from the maximal electroshock threshold assay, while all their WT littermates did (Fisher’s exact test, genotype *p* = 0.014, sex *p* = 0.217). **B** *Scn2a*^*R1627X/+*^ mice were less likely to die from the MES threshold assay than their WT littermates (Chi-square test of independence, genotype *p* = 0.043, sex *p* = 0.413). **C** In the Y84X line, there is no difference in the current at which seizures were induced (two-way ANOVA, genotype *p* = 0.825, sex *p* = 0.688, genotype*sex *p* = 0.429) or (**E**) the severity of the seizures (ranked two-way ANOVA, genotype *p* = 0.575, sex *p* = 0.866, genotype*sex *p* = 0.170). **D** There were no differences in current at which seizures were induced (two-way ANOVA, genotype *p* = 0.076, sex *p* = 0.490, genotype*sex *p* = 0.343) or (**F**) seizure severity in the R1627X line (ranked two-way ANOVA, genotype *p* = 0.167, sex *p* = 0.555, genotype*sex *p* = 0.434). **A-F** Data are expressed as mean ± SEM. **A, C** *n* = 26 (WTs: *n* = 8 males, *n* = 6 females; *Scn2a*^*Y84X/+*^: *n* = 6 males, *n* = 6 females). **B, D, F** *n* = 24 (WTs: *n* = 6 males, *n* = 5 females; *Scn2a*^*R1627X/+*^: *n* = 6 males, *n* = 7 females). **E** *n* = 24 (WTs: *n* = 6 males, *n* = 6 females; *Scn2a*^*Y84X/+*^: *n* = 6 males, *n* = 6 females).

Continuous EEGs were recorded in *Scn2a* PTC mice to evaluate whether they were prone to spontaneous seizures. We observed rare epileptiform activity in both WT and *Scn2a* PTC mice, with no obvious differences in frequency between genotypes in either mouse line, suggesting that *Scn2a* PTC mice do not display more spontaneous seizure activity than WT littermates. To determine whether Na_V_1.2 deficiency increases mortality following seizure induction, we performed a maximal electroshock (MES) threshold assay. In the Y84X line, we observed a main effect of genotype on mortality, such that fewer *Scn2a*^*Y84X/+*^ mice died from the MES assay compared to WT mice (Fig. 5A). Similarly, *Scn2a*^*R1627X/+*^ mice were also less likely to die from the MES assay than their WT littermates (Fig. 5B). We did not observe a main effect of sex in either line. The difference in mortality is not attributable to the current used, as there was no difference between groups for the current that induced a seizure (Fig. 5C-D) nor in the likelihood of remaining seizure-free (Figure S4A-B). Further, we analyzed extension-to-flexion (E/F) ratio, which is an indication of seizure severity [40], and no differences were noted between *Scn2a* PTC mice compared to WT littermates (Fig. 5E-F, Supplementary Table 8). These results suggest that both the Y84X and the R1627X mutations are protective against seizure fatality in the C57BL/6 J genetic background, but do not affect seizure threshold or severity.

## Discussion

Na_V_1.2 supports important roles in neuronal function and has been linked to multiple neuropsychiatric disorders. A central unresolved question in *SCN2A*-related neurodevelopmental disorders is whether distinct loss-of-function variants produce equivalent molecular and cellular outcomes, or whether the specific nature and position of each variant generates allele-specific pathophysiology. In this study, we characterized transcriptional, biochemical, cellular, and behavioral phenotypes associated with two patient-derived PTC variants in *SCN2A* with two newly generated mouse models: *Scn2a*^*Y84X/+*^ and *Scn2a*^*R1627X/+*^. We demonstrate that the location of the PTC governs molecular handling and produces allele-specific physiological and, to a subtler extent, behavioral outcomes.

At the mRNA level, *Scn2a*^*Y84X/+*^-expressing neurons exhibited partial NMD (mutant-allele fraction = 22.5%), a notable finding indicating that an early coding-sequence PTC in *Scn2a* is not fully degraded in the brain (Fig. 1C). Canonical NMD is most efficiently triggered by PTCs positioned more than ~50–55 nucleotides upstream of the last exon-exon junction, and early coding-sequence PTCs are generally considered strong NMD substrates [41]. The incomplete degradation observed here suggests that NMD efficiency in neuronal tissue may be lower than in non-neuronal systems, consistent with reports that neurons exhibit attenuated NMD activity relative to other cell types [42, 43]. In contrast, R1627X transcripts were at allelic balance, consistent with a C-terminal-exon PTC that escapes exon-junction–complex–dependent NMD [41]. Despite these RNA differences, Na_V_1.2 protein was similarly reduced in both lines (~57% of WT) (Fig. 1E). Along with the observation that Na_V_1.2 is maintained significantly above 50% of WT (rather than a strict 1:1 loss), this convergence at the proteome level could suggest site-specific read-through at each PTC or possible compensatory upregulation of the WT allele.

Previous work has shown that *Scn2a* haploinsufficiency disrupts dendritic excitability and synaptic function in mPFC layer 5b pyramidal tract neurons [5, 34], which makes them an ideal model for dissecting the consequences of *Scn2a* premature stop codons via current-clamp recordings. Physiologically, our findings indicate that a seemingly equivalent reduction of protein can produce non-equivalent functional outcomes. This challenges the assumption that Na_V_1.2 haploinsufficiency is a uniform disease state. Despite comparable total Na_V_1.2 abundance, AP upstroke velocity was more severely attenuated in Y84X than in R1627X-expressing neurons (26.7% vs 10.3%, respectively) (Fig. 2C). Moreover, spike threshold is depolarized only in *Scn2a*^*Y84X/+*^ mice, whereas *Scn2a*^*R1627X/+*^ neuron thresholds remain indistinguishable from WT (Fig. 2D). Spike threshold depolarization is observed in *Scn2a* heterozygous mice, but only at very young ages (P4-P8) and is indistinguishable from WT mice by P10 [5], such that Y84X-expressing neurons appear physiologically immature compared to WT and R1627X-expressing neurons by this metric. One possibility is that the partial NMD of Y84X mRNA may permit translation of a short N-terminal peptide fragment that could alter AIS channel organization through an unknown mechanism. Alternatively, while *Scn2a*^*R1627X/+*^ neurons could retain (or recruit) compensatory Na_V_1.6-mediated AIS excitability to preserve AP threshold, this mechanism is apparently less effective in Y84X neurons. Under those models, similar reductions in bulk Na_V_1.2 abundance would produce distinct electrophysiological outcomes because AP firing is governed not simply by total sodium channel content, but by isoform-specific channel composition and distribution at the AIS. Either mechanistic possibility presents a significant experimental challenge for future studies but provides a novel framework to account for the diverse phenotypes of people harboring *SCN2A* PTC variants.

In this framework, the Y84X variant exerts a greater impact on somatic upstroke kinetics and rheobase-proximal firing. These allele-dependent signatures reinforce that PTC context — not merely the presence of a PTC — governs how Na_V_1.2 haploinsufficiency manifests at the level of neuronal physiology.

### Behavioral phenotypes associated with *Scn2a* PTCs

Both *Scn2a*^*Y84X/+*^ and *Scn2a*^*R1627X/+*^ mice showed mild reductions in body weight compared to their respective WT controls. Gross motor activity was preserved across genotypes, and neither line showed impairments in baseline locomotion, similar to some haploinsufficient *Scn2a* models, although hyperactivity has also been reported [44–46]. In contrast, *Scn2a*^*Y84X/+*^ mice showed a male-specific impairment in motor learning on the rotarod task, a phenotype reported in one other model [46] but not observed in *Scn2a*^*R1627X/+*^ mice or most other haploinsufficient models [5, 44, 45]. This variant and sex-specific impairment may reflect subtle differences in cerebellar function, due to Na_V_1.2’s expression in cerebellar granule neurons [36].

Additionally, *Scn2a*^*Y84X/+*^ mice spent significantly more time in the open arms of the elevated zero maze, suggesting increased risk-taking behavior, while *Scn2a*^*R1627X/+*^ mice showed no alterations. Anecdotally, a few *Scn2a*^*Y84X/+*^ and *Scn2a*^*R1627X/+*^ mice had unusual exploratory behaviors, such as jumping or diving from the elevated zero maze, or male *Scn2a*^*Y84X/+*^ mice jumping out of the arena in the three-chamber social task. Instances of increased exploratory behavior have been observed elsewhere [5, 45, 46], primarily in the elevated zero or elevated plus maze. Similar to our findings, most models show no differences in the open field test [5, 44, 45]. These increased exploration phenotypes could reflect sensory-seeking behaviors, although more careful experimentation focused on sensory function and risk-taking behaviors are required to clarify this.

A key finding was the increase in grooming behaviors in both *Scn2a*^*Y84X/+*^ and *Scn2a*^*R1627X/+*^ mice, suggesting increased repetitive and restrictive behavior – a core diagnostic feature of ASD. Both *Scn2a*^*Y84X/+*^ and *Scn2a*^*R1627X/+*^ mice spent more time grooming, and *Scn2a*^*R1627X/+*^ males showed higher grooming frequency than WT controls. The long time spent in a single grooming bout suggests behavioral perseveration. Increased grooming behavior has been reported in some juvenile *Scn2a* haploinsufficient models, but not in adults except for the gene-trap model [5, 44, 45, 47, 48]. Our findings suggest that Na_V_1.2 deficiency increases repetitive behaviors into adulthood. Other domains—sociability in the three-chamber task and sensorimotor gating—were intact in both lines, suggesting a selective rather than global alteration of behavior.

Finally, in the MES assay, both *Scn2a*^*Y84X/+*^ and *Scn2a*^*R1627X/+*^ mice showed reduced seizure-induced mortality, despite having similar seizure thresholds and severity compared to WT littermates. While this superficially suggests that *Scn2a* PTC variants confer protection against fatal seizures, children with *SCN2A* LOF mutations often develop seizure disorders, including the child documented with a p.Y84X mutation on ClinVar (see link in Supplemental Information), which can be difficult to treat or fatal. Seizures have been evaluated in other *Scn2a* haploinsufficient models [49, 50], with two reporting epileptiform activity and absence-like seizures not observed in our mouse models [29, 51]. The genetic strain used for both of our *Scn2a* PTC lines, C57BL/6 J, is associated with less severe seizure phenotypes in many models of SCN2A-related disorders [4, 25–27, 29, 50, 52], so this particular result should be interpreted with caution.

Together, the behavioral data demonstrate that the Y84X and R1627X variants result in overlapping but allele-specific phenotypes. *Scn2a*^*Y84X/+*^ mice show more pronounced changes—mirrored by stronger alterations in slice physiology—in motor and exploratory behaviors. Both *Scn2a*^*Y84X/+*^ and *Scn2a*^*R1627X/+*^ mice display restrictive and repetitive grooming and appear protected against MES-induced mortality. However, only *Scn2a*^*Y84X/+*^ mice display increased exploration and locomotor learning deficits. Overall, these findings demonstrate that distinct PTC variants are not interchangeable, producing partially shared but allele-specific functional outcomes across molecular, cellular, and behavioral levels.

### Challenges, limitations, and future directions

We assessed mRNA and protein expression using whole brain lysates, precluding cell type-specific analyses. NMD and Na_V_1.2 levels may differ in mPFC pyramidal tract neurons versus other neuronal types; cell type-specific measurements would help resolve allele-specific mechanisms. We did not quantify Na_V_1.2 protein levels in the mice that underwent behavioral testing, ruling out subject-level protein-phenotype correlations. Targeted Na_V_1.2 quantification in behavior-tested cohorts would be needed to assess causality. As with any mouse model, behavioral assays provide construct—not symptom—validity, and results should not be over-interpreted given the heterogeneity and multi-faceted nature of ASD and ID symptomology in humans. Finally, we behaviorally tested mice between 16–30 weeks of age, which is roughly equivalent to mid-adulthood in humans. Since *SCN2A* is important for early neuronal maturation [4], future work should include observations earlier in development.

## Supplementary information

Supplementary Material

## Data Availability

The data that support the findings of this study are available on request from the corresponding authors.

## References

[1] Catterall WA. From ionic currents to molecular mechanisms. Neuron. 2000;26:13–25. 10.1016/S0896-6273(00)81133-2 10798388

[2] Catterall WA. Structure and function of voltage‐gated sodium channels at atomic resolution. Exp Physiol. 2014;99:35–51. 10.1113/expphysiol.2013.071969 PMC3885250 24097157

[3] Hu W, Tian C, Li T, Yang M, Hou H, Shu Y. Distinct contributions of Nav1.6 and Nav1.2 in action potential initiation and backpropagation. Nat Neurosci. 2009;12:996–1002. 10.1038/nn.2359 19633666

[4] Sanders SJ, Campbell AJ, Cottrell JR, Moller RS, Wagner FF, Auldridge AL, et al. Progress in understanding and treating SCN2A-Mediated disorders. Trends Neurosci. 2018;41:442–56. 10.1016/j.tins.2018.03.011 PMC6015533 29691040

[5] Spratt PWE, Ben-Shalom R, Keeshen CM, Burke KJ, Clarkson RL, Sanders SJ, et al. The autism-associated gene scn2a contributes to dendritic excitability and synaptic function in the prefrontal cortex. Neuron. 2019;103:673–685.e5. 10.1016/j.neuron.2019.05.037 PMC7935470 31230762

[6] Sanders SJ, Murtha MT, Gupta AR, Murdoch JD, Raubeson MJ, Willsey AJ, et al. De novo mutations revealed by whole-exome sequencing are strongly associated with autism. Nature. 2012;485:237–41. 10.1038/nature10945 PMC3667984 22495306

[7] Ben-Shalom R, Keeshen CM, Berrios KN, An JY, Sanders SJ, Bender KJ. Opposing effects on Na V 1.2 function underlie differences between SCN2A variants observed in individuals with autism spectrum disorder or infantile seizures. Biol Psychiatry. 2017;82:224–32. 10.1016/j.biopsych.2017.01.009 PMC5796785 28256214

[8] Ogiwara I, Ito K, Sawaishi Y, Osaka H, Mazaki E, Inoue I, et al. De novo mutations of voltage-gated sodium channel α_II_ gene *SCN2A* in intractable epilepsies. Neurology. 2009;73:1046–53. 10.1212/WNL.0b013e3181b9cebc PMC2754324 19786696

[9] Liao Y, Anttonen AK, Liukkonen E, Gaily E, Maljevic S, Schubert S, et al. *SCN2A* mutation associated with neonatal epilepsy, late-onset episodic ataxia, myoclonus, and pain. Neurology. 2010;75:1454–8. 10.1212/WNL.0b013e3181f8812e 20956790

[10] Shi X, Yasumoto S, Kurahashi H, Nakagawa E, Fukasawa T, Uchiya S, et al. Clinical spectrum of SCN2A mutations. Brain Dev. 2012;34:541–5. 10.1016/j.braindev.2011.09.016 22029951

[11] Adney SK, Millichap JJ, DeKeyser J, Abramova T, Thompson CH, George AL. Functional and pharmacological evaluation of a novel *SCN2A* variant linked to early‐onset epilepsy. Ann Clin Transl Neurol. 2020;7:1488–501. 10.1002/acn3.51105 PMC7480906 32750235

[12] Li J, Cai T, Jiang Y, Chen H, He X, Chen C, et al. Genes with de novo mutations are shared by four neuropsychiatric disorders discovered from NPdenovo database. Mol Psychiatry. 2016;21:290–7. 10.1038/mp.2015.40 PMC4837654 25849321

[13] Begemann A, Acuña MA, Zweier M, Vincent M, Steindl K, Bachmann-Gagescu R, et al. Further corroboration of distinct functional features in SCN2A variants causing intellectual disability or epileptic phenotypes. Mol Med. 2019;25:6. 10.1186/s10020-019-0073-6 PMC6391808 30813884

[14] Murray M, Martindale JM, Otallah SI. *SCN2A* - Associated episodic and persistent ataxia with cerebellar atrophy: a case report. Child Neurol Open. 2023;10:2329048X231163944. 10.1177/2329048X231163944 PMC10026144 36950068

[15] Zandi PP, Jaffe AE, Goes FS, Burke EE, Collado-Torres L, Huuki-Myers L, et al. Amygdala and anterior cingulate transcriptomes from individuals with bipolar disorder reveal downregulated neuroimmune and synaptic pathways. Nat Neurosci. 2022;25:381–9. 10.1038/s41593-022-01024-6 PMC8915427 35260864

[16] Mullins N, Forstner AJ, O’Connell KS, Coombes B, Coleman JRI, Qiao Z, et al. Genome-wide association study of more than 40,000 bipolar disorder cases provides new insights into the underlying biology. Nat Genet. 2021;53:817–29. 10.1038/s41588-021-00857-4 PMC8192451 34002096

[17] eQTLGen Consortium, BIOS Consortium, the Bipolar Disorder Working Group of the Psychiatric Genomics Consortium, Stahl EA, Breen G, Forstner AJ, et al. Genome-wide association study identifies 30 loci associated with bipolar disorder. Nat Genet. 2019;51:793–803. 10.1038/s41588-019-0397-8 PMC6956732 31043756

[18] Dickinson D, Straub RE, Trampush JW, Gao Y, Feng N, Xie B, et al. Differential effects of common variants in *SCN2A* on general cognitive ability, brain physiology, and messenger rna expression in schizophrenia cases and control individuals. JAMA Psychiatry. 2014;71:647. 10.1001/jamapsychiatry.2014.157 PMC4160812 24718902

[19] Fromer M, Pocklington AJ, Kavanagh DH, Williams HJ, Dwyer S, Gormley P, et al. De novo mutations in schizophrenia implicate synaptic networks. Nature. 2014;506:179–84. 10.1038/nature12929 PMC4237002 24463507

[20] Carroll LS, Woolf R, Ibrahim Y, Williams HJ, Dwyer S, Walters J, et al. Mutation screening of SCN2A in schizophrenia and identification of a novel loss-of-function mutation. Psychiatr Genet. 2016;26:60–5. 10.1097/YPG.0000000000000110 PMC4756433 26555645

[21] Thompson CH, Potet F, Abramova TV, DeKeyser JM, Ghabra NF, Vanoye CG, et al. Epilepsy-associated *SCN2A* (NaV1.2) variants exhibit diverse and complex functional properties. J Gen Physiol. 2023;155:e202313375. 10.1085/jgp.202313375 PMC10424433 37578743

[22] Planells-Cases R, Caprini M, Zhang J, Rockenstein EM, Rivera RR, Murre C, et al. Neuronal death and perinatal lethality in voltage-gated sodium channel αii-deficient mice. Biophys J. 2000;78:2878–91. 10.1016/S0006-3495(00)76829-9 PMC1300874 10827969

[23] Kamiya K, Kaneda M, Sugawara T, Mazaki E, Okamura N, Montal M, et al. A nonsense mutation of the sodium channel gene *SCN2A* in a patient with intractable epilepsy and mental decline. J Neurosci. 2004;24:2690–8. 10.1523/JNEUROSCI.3089-03.2004 PMC6729532 15028761

[24] Lindeboom RGH, Vermeulen M, Lehner B, Supek F. The impact of nonsense-mediated mRNA decay on genetic disease, gene editing and cancer immunotherapy. Nat Genet. 2019;51:1645–51. 10.1038/s41588-019-0517-5 PMC6858879 31659324

[25] Scott KEJ, Hermosillo Arrieta MF, Williams AJ. Deciphering *SCN2A* : a comprehensive review of rodent models of *Scn2a* dysfunction. Epilepsia. 2025;66:4624–4638. 10.1111/epi.18615 PMC12779317 40884529

[26] Schattling B, Fazeli W, Engeland B, Liu Y, Lerche H, Isbrandt D, et al. Activity of NaV1.2 promotes neurodegeneration in an animal model of multiple sclerosis. JCI Insight. 2016;1:e89810. 10.1172/jci.insight.89810 PMC5111507 27882351

[27] Echevarria-Cooper DM, Hawkins NA, Misra SN, Huffman AM, Thaxton T, Thompson CH, et al. Cellular and behavioral effects of altered NaV1.2 sodium channel ion permeability in *Scn2a K1422E* mice. Hum Mol Genet. 2022;31:2964–88. 10.1093/hmg/ddac087 PMC9433730 35417922

[28] Li M, Eltabbal M, Tran HD, Kuhn B. Scn2a insufficiency alters spontaneous neuronal Ca2+ activity in somatosensory cortex during wakefulness. iScience. 2023;26:108138. 10.1016/j.isci.2023.108138 PMC10590963 37876801

[29] Ogiwara I, Miyamoto H, Tatsukawa T, Yamagata T, Nakayama T, Atapour N, et al. Nav1.2 haplodeficiency in excitatory neurons causes absence-like seizures in mice. Commun Biol. 2018;1:96. 10.1038/s42003-018-0099-2 PMC6115194 30175250

[30] Clarkson RL, Liptak AT, Gee SM, Sohal VS, Bender KJ. D3 receptors regulate excitability in a unique class of prefrontal pyramidal cells. J Neurosci. 2017;37:5846–60. 10.1523/JNEUROSCI.0310-17.2017 PMC5473204 28522735

[31] Friard O, Gamba M BORIS : a free, versatile open‐source event‐logging software for video/audio coding and live observations. Fitzjohn R, editor. Methods Ecol Evol. 2016;7:1325–30. 10.1111/2041-210X.12584

[32] Petrucci AN, Joyal KG, Chou JW, Li R, Vencer KM, Buchanan GF. Post-ictal generalized EEG suppression is reduced by enhancing dorsal raphe serotonergic neurotransmission. Neuroscience. 2021;453:206–21. 10.1016/j.neuroscience.2020.11.029 PMC8713886 33242541

[33] Lee AT, Gee SM, Vogt D, Patel T, Rubenstein JL, Sohal VS. Pyramidal neurons in prefrontal cortex receive subtype-specific forms of excitation and inhibition. Neuron. 2014;81:61–8. 10.1016/j.neuron.2013.10.031 PMC3947199 24361076

[34] Spratt PWE, Alexander RPD, Ben-Shalom R, Sahagun A, Kyoung H, Keeshen CM, et al. Paradoxical hyperexcitability from NaV1.2 sodium channel loss in neocortical pyramidal cells. Cell Rep. 2021;36:109483. 10.1016/j.celrep.2021.109483 PMC8719649 34348157

[35] Garcia JD, Wang C, Alexander RP, Banks E, Fenton T, DeKeyser JM, et al. Differential roles of NaV1.2 and NaV1.6 in neocortical pyramidal cell excitability. eLife. 2025;14:RP105696. 10.7554/eLife.105696 PMC12263156 40662949

[36] Martínez-Hernández J, Ballesteros-Merino C, Fernández-Alacid L, Nicolau JC, Aguado C, Luján R. Polarised localisation of the voltage-gated sodium channel Nav1.2 in cerebellar granule cells. Cerebellum. 2013;12:16–26. 10.1007/s12311-012-0387-1 22528969

[37] American Psychiatric Association. Diagnostic and statistical manual of mental disorders. Fifth Edition. American Psychiatric Association; 2013. https://psychiatryonline.org/doi/book/10.1176/appi.books.9780890425596.

[38] Cheng CH, Chan PYS, Hsu SC, Liu CY. Meta-analysis of sensorimotor gating in patients with autism spectrum disorders. Psychiatry Res. 2018;262:413–9. 10.1016/j.psychres.2017.09.016 28918862

[39] Wang C, Derderian KD, Hamada E, Zhou X, Nelson AD, Kyoung H, et al. Impaired cerebellar plasticity hypersensitizes sensory reflexes in SCN2A-associated ASD. Neuron. 2024;112:1444–1455.e5. 10.1016/j.neuron.2024.01.029 PMC11065582 38412857

[40] Anderson RE, Howard RA, Woodbury DM. Correlation between effects of acute acetazolamide administration to mice on electroshock seizure threshold and maximal electroshock seizure pattern, and on carbonic anhydrase activity in subcellular fractions of brain. Epilepsia. 1986;27:504–9. 10.1111/j.1528-1157.1986.tb03575.x 3093209

[41] Nagy E, Maquat LE. A rule for termination-codon position within intron-containing genes: when nonsense affects RNA abundance. Trends Biochem Sci. 1998;23:198–9. 10.1016/S0968-0004(98)01208-0 9644970

[42] Bruno IG, Karam R, Huang L, Bhardwaj A, Lou CH, Shum EY, et al. Identification of a MicroRNA that activates gene expression by repressing nonsense-mediated RNA decay. Mol Cell. 2011;42:500–10. 10.1016/j.molcel.2011.04.018 PMC3123134 21596314

[43] Lin L, Zhang M, Stoilov P, Chen L, Zheng S. Developmental attenuation of neuronal apoptosis by neural-specific splicing of bak1 microexon. Neuron. 2020;107:1180–1196.e8. 10.1016/j.neuron.2020.06.036 PMC7529960 32710818

[44] Léna I, Mantegazza M. NaV1.2 haploinsufficiency in Scn2a knock-out mice causes an autistic-like phenotype attenuated with age. Sci Rep. 2019;9:12886. 10.1038/s41598-019-49392-7 PMC6733925 31501495

[45] Marcantonio W, Simonti M, Léna I, Mantegazza M. Sex-specific behavioral features of juvenile and adult haploinsufficient Scn2a+/- female mice, model of autism spectrum disorder. Genes Brain Behav. 2025;24:e70034. 10.1111/gbb.70034 PMC12399408 40887751

[46] Tatsukawa T, Raveau M, Ogiwara I, Hattori S, Miyamoto H, Mazaki E, et al. Scn2a haploinsufficient mice display a spectrum of phenotypes affecting anxiety, sociability, memory flexibility and ampakine CX516 rescues their hyperactivity. Mol Autism. 2019;10:15. 10.1186/s13229-019-0265-5 PMC6437867 30962870

[47] Indumathy J, Pruitt A, Gautier NM, Crane K, Glasscock E. Kv1.1 deficiency alters repetitive and social behaviors in mice and rescues autistic‐like behaviors due to *Scn2a* haploinsufficiency. Brain Behav. 2021;11:e02041. 10.1002/brb3.2041 PMC8035482 33484493

[48] Eaton M, Zhang J, Ma Z, Park AC, Lietzke E, Romero CM, et al. Generation and basic characterization of a gene‐trap knockout mouse model of *Scn2a* with a substantial reduction of voltage‐gated sodium channel Na_v_ 1.2 expression. Genes Brain Behav. 2021;20:e12725. 10.1111/gbb.12725 33369088

[49] Mishra V, Karumuri BK, Gautier NM, Liu R, Hutson TN, Vanhoof-Villalba SL, et al. Scn2a deletion improves survival and brain–heart dynamics in the Kcna1-null mouse model of sudden unexpected death in epilepsy (SUDEP). Hum Mol Genet. 2017;26:2091–103. 10.1093/hmg/ddx104 PMC6075631 28334922

[50] Schamiloglu S, Wu H, Zhou M, Kwan AC, Bender KJ. Dynamic foraging behavior performance is not affected by *Scn2a* haploinsufficiency. eneuro. 2023;10:ENEURO.0367-23.2023 10.1523/ENEURO.0367-23.2023 PMC10755640 38151324

[51] Miyamoto H, Tatsukawa T, Shimohata A, Yamagata T, Suzuki T, Amano K, et al. Impaired cortico-striatal excitatory transmission triggers epilepsy. Nat Commun. 2019;10:1917 10.1038/s41467-019-09954-9 PMC6478892 31015467

[52] Echevarria-Cooper DM, Hawkins NA, Kearney JA Strain-dependent effects on neurobehavioral and seizure phenotypes in *Scn2a*^*K1422E*^ mice. Neuroscience [Preprint]. 2023 [cited 2025 Sep 15]. Available from: http://biorxiv.org/lookup/doi/10.1101/2023.06.06.543929.

