# Supplementary material for: Nonsense-mediated decay influences position-dependent effects of SCN2A premature stop codons on neuronal excitability and behavior
Source: Mol Psychiatry. 2026 Jul 6;31(11):6547–58. doi: 10.1038/s41380-026-03723-z (PMC13668693; doi:10.1038/s41380-026-03723-z)
Supplement: Supplementary file 1 [file 41380_2026_3723_MOESM1_ESM.pdf]

Supplementary Material

Al Saneh et al, *Nonsense mediated decay influences position-dependent effects of SCN2A premature stop codons on neuronal excitability and behavior*

Transgenic mouse generation

The Y84X and R1627X mutations are found in *SCN2A* patients (1) (Y84X: https://www.ncbi.nlm.nih.gov/clinvar/variation/VCV000984819.2; R1626X: https://www.ncbi.nlm.nih.gov/clinvar/variation/410985). Due to a single amino acid insertion in the mouse *Scn2a* gene, R1627X in mice is equivalent to R1626X in humans. Transgenic mice were generated in collaboration with the Iowa Genome Editing Core using CRISPR/Cas12 (*Scn2a*$^{Y84X/+}$) and CRISPR/Cas9 (*Scn2a*$^{R1627X/+}$). Both lines were generated on the C57BL/6J strain from Jackson Labs (Strain #000664, Bar Harbor, ME). Male mice older than 8 weeks were bred with 3 to 5-week-old super-ovulated females to produce zygotes for pronuclear injection. Female ICR (Envigo; Hsc:ICR(CD-1)) mice were used as recipients for embryo transfer.

For the *Scn2a*$^{Y84X/+}$ mice, chemically modified CRISPR-Cas12a crRNAs were purchased from IDT (Alt-R™ A.s. Cas12a crRNA). crRNA sequences were TTATTGATATAGTAGGGGTCCAG and TGAAAAGAGGCACCCTCTTCACCATCTTAAAGAAATCGAGGAAATAAAAGCAACTGG TCTAAAAACTCACCTTCTTATTGATGTATTATGGATCCAGGTCCTCCAGGGGCTCTGAC ACCATCTCTGGAGGAATGTCTCCATAAATAAAAGGAAGGGATTTTCCTGC. The crRNAs were suspended in T10E0.1 buffer at 1 µg/µl, heated at 98°C for 2 min, and allowed to cool slowly to 20°C in a thermal cycler. The annealed crRNAs were aliquoted into single-use tubes and stored at -80°C. Cas12a nuclease was also purchased from IDT (Alt-R™ A.s. Cas12a (Cpf1) V3; Catalog #1081068). Cas12a crRNA ribonucleoprotein complexes were made by combining Cas12a protein and crRNA in T10E0.1 (final concentrations: 1190 ng/µl (~7.3 µM) Cas12a protein and 760 ng/µl (~22.4 µM) crRNA). The Cas12a protein and annealed RNAs were incubated at 37°C for 10 min. The RNP complexes were combined with single-stranded oligonucleotide repair template and incubated an additional 5 min at 37°C. The final concentrations in the injection mix were 100 ng/µl (~0.6 µM) Cas12a protein, 64 ng/µl (~1.9 µM) crRNA, and 500 ng/µl oligonucleotide repair template. Pronuclear-stage embryos were collected using methods described elsewhere (2).

Embryos were collected in KSOM media (Millipore; MR101D) and washed 3 times to remove cumulus cells. Cas12a RNPs and single-stranded repair templates were injected (3) into the pronuclei of the collected zygotes. Cas12a RNPs and oligonucleotide repair template were electroporated into zygotes briefly treated (10-sec) acid Tyrode's solution (Sigma; T1788). The embryos were then washed 5 times with Opti-MEM, and 25-30 were aligned between the electrodes of a slide glass plate electrode with a 1.0 mm gap and platinum leads. The acid-treated embryos were overlaid with the electroporation mix and electroporated for 11 pulses at 30V (1-msec on:100-msec off). Electroporation was performed using an ECM830 square wave electroporator (BTX). Zygotes from both were incubated in KSOM with amino acids at 37°C under 5% CO_2 until all zygotes were ready for injection. Fifteen to 25 embryos were immediately implanted into the oviducts of pseudo-pregnant ICR females.

For the *Scn2a*$^{R1627X/+}$ mice, chemically modified CRISPR-Cas9 crRNAs and CRISPR-Cas9 tracrRNA were purchased from IDT (Alt-R® CRISPR-Cas9 crRNA; Alt-R® CRISPR-Cas9 tracrRNA (Cat# 1072532)). crRNA sequences were Scn2a_R1627stop_5PB ATCTTACAAACTGCACATAT and Scn2a_R1627stop_3PD TTTTCAGGGTCACAATCGGG. The crRNAs and tracrRNA were suspended in T10E0.1 buffer and combined to 1 µg/µl (~29.5 µM) final concentration in a 1:2 (µg:µg) ratio. The RNAs were heated at 98°C for 2 min and allowed to cool slowly to 20°C in a thermal cycler. The annealed cr:tracrRNAs were aliquoted to single-use tubes and stored at -80°C. Cas9 nuclease was also purchased from IDT (Alt-R® S.p. HiFi Cas9 Nuclease). Cr:tracr:Cas9 ribonucleoprotein complexes were made by combining Cas9 protein and cr:tracrRNA in T10E0.1 (final concentrations: 300 ng/µl (~1.9 µM) Cas9 protein and 200 ng/µl (~5.9 µM) cr:tracrRNA). The Cas9 protein and annealed RNAs were incubated at 37°C for 10 min. The RNP complexes were combined with single-stranded repair template and incubated an additional 5 min at 37°C. The final concentrations in the injection mix were 60 ng/µl (~0.4 µM) Cas9 protein, 10 ng/µl (~0.3 µM) each cr:tracrRNA, and 20 ng/µl single-stranded repair template. Pronuclear-stage embryos were collected using methods described in (2). Embryos were collected in KSOM media (Millipore; MR101D) and washed 3 times to remove cumulous cells. Cas9 RNPs and single-stranded repair template were injected (3) into the pronuclei of the collected zygotes and incubated in KSOM with amino acids at 37°C under 5% CO_2 until all zygotes were injected. Fifteen to 25 embryos were immediately implanted into the oviducts of pseudo-pregnant ICR females.

Both mouse lines were backcrossed onto the C57BL/6J background for at least four generations before incorporation into the study. To generate experimental animals, *Scn2a*$^{Y84X/+}$ or *Scn2a*$^{R1627X/+}$ mice were bred to WT mice to produce litters with combinations of *Scn2a*$^{Y84X/+}$ and WT offspring (or *Scn2a*$^{R1627X/+}$ and WT offspring, respectively). The lines were bred and maintained separately and transgenic mice were cohoused with their own WT littermates. Pups were weaned 21–24 days after birth.

Genotyping

Mice were genotyped at weaning via ear punch, and genotypes were confirmed via tail snips post-mortem. Genomic DNA (gDNA) was extracted with a tissue DNA lysate (NaOH and EDTA in ddH_2O) and neutralization buffer (Tris-HCl in ddH_2O). Polymerase chain reaction (PCR) was then performed on gDNA along with the following primers: Y84X Scn2a_Tyr84TAA:F:5′GAACAACAGGCTCAACCAAATG3′ and Scn2a_Tyr84TAA:R:5′GGGAATCCCTTGCTGCTATT3′; or R1627X LG366_R1627:F:5’CCCACCCTCTGATGGTCTTA3’ and LG236_Scn2a_R1627:R:5’ACCACAACCAGGAAGGATATG3’), GoTaq(R) G2 Green Master Mix (Catalog #M7823, Promega, Madison, WI), and ddH_2O. PCR conditions for the Y84X line were 95ºC for 2 min, then 35 cycles of 95ºC for 25 sec, 62ºC for 25 sec, and 72ºC for 1 min 15 sec, and a final inactivation step of 72ºC for 5 min. For the R1627X line, PCR conditions were 95ºC for 2 min, then 37 cycles of 98ºC for 20 sec, 61ºC for 30 sec, and 72ºC for 2 min, and a final inactivation step of 72ºC for 7 min. The PCR product was then digested with a 1:1 ratio of BamHI-HF and rCutSmart buffer for the Y84X line (Catalog #R3136S, New England Biolabs, Ipswich, MA), or a 1:1 ratio of DdeI and rCutSmart buffer for the R1627X line (Catalog #R0175S, New England Biolabs) at 37ºC for 45 min. Following enzyme restriction, the samples were separated by electrophoresis on a 2% agarose gel.

Statistics

Data were graphed and analyzed using OriginPro (OriginLab, Northampton, MA), GraphPad Prism 10.5 (GraphPad Software; San Diego, CA) and RStudio (R4.4.0, afex 1.4-1, emmeans 1.11.1, lme4 1.1-37, lmerTest 3.1-3, rcompanion 2.5.0, survival 3.8-3). Data were analyzed using

the statistical tests noted in the results and figure legends (two-way ANOVA, ranked two-way ANOVA, Fisher's exact test, Chi-square test of independence, log-rank test, linear mixed effects model, or linear model with appropriate follow-up testing).

Nonsense-Mediated Decay

Purified RNA was converted to cDNA using the High-Capacity cDNA Reverse Transcription Kit (Thermo Fisher Scientific). Locus-specific PCR was then performed to amplify (i) exons 1–6 of Scn2a from *Scn2a*$^{Y84X/+}$ samples (capturing codon 84) and (ii) exon 27 from *Scn2a*$^{R1627X/+}$ samples (capturing codon 1627). PCR products were purified and Sanger sequenced by Azenta Life Sciences (South Plainfield, NJ), and amplicon next-generation sequenced by Plasmidsaurus (Louisville, KY) for allele-fraction quantification. Sample sizes were insufficient to examine a genotype-by-sex interaction in *Scn2a* transcript abundance by two-way ANOVA; however, visual inspection of the data did not reveal an apparent sex-dependent difference.

Source of Variation	% of total variation	P value	P value summary	Significant?
Interaction	0.6898	0.1412	ns	No
Sex	0.005840	0.8476	ns	No
Genotype	88.53	0.0014	**	Yes

Sanger sequencing Primers

Scn2a$^{Y84X/+}$ mouse samples: AAATGGCCCAAAGCCAAATAG

Scn2a$^{R1627X/+}$ mouse samples: CATCTCAAAGTCGTCCTCACTC

PCR amplification Primers

Scn2a$^{Y84X/+}$ mouse samples:

Forward Primer: CAAGGACGAAGACGACGAAA

Reverse Primer: GGCAGTACCATTCCAATCCA

Scn2a$^{R1627X/+}$ mouse samples:

Forward Primer: GCGGAGCTGATAGAGAAGTATT

Reverse Primer 1: CGCTTCAGAGTGGTGGTAAT

Reverse Primer 2: CATAAGAGACCTTGGAGGGATTG

Na_V1.2 protein expression levels

Two-way ANOVA revealed no significant genotype-by-sex interaction for Na_V1.2 protein abundance in *Scn2a*$^{Y84X/+}$and *Scn2a*$^{R1627X/+}$ mice.

Source of Variation	% of total variation	P value	P value summary	Significant?
Interaction	1.053	0.1711	ns	No
Sex	0.4473	0.2140	ns	No
Genotype	85.86	<0.0001	****	Yes

Ex vivo Electrophysiology

After isoflurane anesthesia and rapid brain removal, slices were cut in ice-cold bath solution and allowed to recover > 30 minutes at room temperature and 5% CO_2 before recordings at 32 °C. In acute mPFC slices, corticofugal layer 5b neurons can be distinguished by their high expression of HCN channels that manifest as voltage 'sag' visible during a strong hyperpolarization step (−400 pA, 120 ms), and a rebound that peaks to a more depolarized potential than V_m at rest within 90 ms of step offset (4). Signals were sampled at 50 kHz and low-pass filtered at 20 kHz. Pipette capacitance was compensated to 50% of Cp fast, and bridge balance was applied. Series resistance was <12 MΩ for all included recordings; fast pipette capacitance was <12 pF. Action potentials (APs) were evoked with 300-pA, 300-ms depolarizing steps. For biophysical analyses, AP threshold and the maximum rate of rise (peak dV/dt) were quantified from the first spike in a train. Threshold was defined as the membrane potential at which dV/dt first exceeded 13 V s^{-1}; somatic peak dV/dt was taken as the maximal derivative during the upstroke.

External solution contained (in mM): 125 NaCl, 25 glucose, 21.5 $NaHCO_3$, 1 $MgCl_2 \cdot 6H_2O$, 2.5 KCl, 1.25 NaH_2PO_4 (anhydrous), and 2 $CaCl_2 \cdot 2H_2O$; osmolarity, 302 mOsm. Patch pipettes (Warner Instruments, G150TF-4) were pulled to 3-4 MΩ and filled with (in mM): 113 K-gluconate,

9 HEPES, 4.5 $MgCl_2 \cdot 6H_2O$, 10 sucrose, 14 Tris-phosphocreatine, 4 Na_2-ATP (monohydrate), 0.3 Tris-GTP, and 0.1 EGTA; pH 7.2–7.5; 285 mOsm.

General behavioral procedures

All mice were adults (16-30 weeks) at the time of behavioral testing. Sample sizes are indicated in each figure. Behavioral experiments were run in two independent cohorts of mice in the following order: Cohort 1 underwent elevated zero maze, open field test, rotarod, and electroencephalogram (EEG) and electromyography (EMG) monitoring; Cohort 2 underwent three-chamber social task, prepulse inhibition, and maximal electroshock (MES) seizure induction. A minimum of 2 days was taken between every behavioral assay to reduce the impact of stress from previous tests on subsequent tests. Mice were randomized and color-coded with Sharpie markers on their tails at the beginning of behavioral testing to maintain blinding during testing.

Elevated Zero Maze

Mice were placed in a beige ABS plastic maze (San Diego Instruments, San Diego, CA) elevated 50.0 cm above the table, with an internal diameter of 50.8 cm and an outer diameter of 60.96 cm (internal pathway 5.0 cm wide). The walls on the closed sections were 15.24 cm, and the lip on the open sections was 1.0 cm high. Above the maze, a 45.7 cm ring light (Inkeltech, HaiNing Da Mai E-Commerce Co., China) was positioned, resulting in ~245 lux in the open sections of the maze. Each mouse underwent a single 5 min trial. Activity was tracked using EthoVision XT 17 software (Noldus, Wageningen, The Netherlands) for distance traveled, velocity, and duration spent in open/closed sections. Mice were excluded from the analysis if they failed to stay on the maze for the entire duration of the trial.

Open Field Test

Mice were placed in a custom TAP black plastic arena (L × W × H = 40.0 cm × 42.0 cm × 30.0 cm) over a P95 matte acrylic table. A 45.7 cm ring light was mounted overhead, resulting in ~115 lux (center) and ~100 lux (corners). Each mouse underwent a single 10 min trial. Activity was tracked by EthoVision XT 17 software for distance traveled, velocity, and thigmotaxis (the tendency to stay at the edge of the arena). For the latter, the arena was divided into the periphery and the center, each comprising 50% of the total surface area of the arena. Grooming was manually

scored by an independent, blind observer from videos with the Behavioral Observation Research Interactive Software (BORIS) (5).

Rotarod

Mice were handled for 3 consecutive days before the assay. During the assay, mice were placed on the rotating drum of an accelerating rotarod (Ugo Basile, Varese, Italy). The rotarod accelerated from 4 to 40 rpm over a 5 min period. Mice were given 3 trials/day for 5 consecutive days, with a maximum trial duration of 5 min and an inter-trial interval of at least 10 min. Latency to fall or second passive rotation was recorded for each mouse each day.

Three-Chamber Social Task

Mice were placed in a matte, black plastic rectangular arena (L × W × H = 51.0 cm × 25.4 cm × 25.4 cm) divided into three compartments, with an 11.0 cm wide opening between compartments and one empty, clear acrylic perforated cylinder in the center of each outer compartment. Mice were first allowed to habituate to the arena for 10 min. Following habituation, mice were placed in a clean cage, while experimenters placed a novel mouse, matched for strain, sex, age, and weight (+/- 5.0 g), under one cylinder and a novel, plastic block under the other. Placement of the novel object and novel mice was randomized. After, the experiment mice were allowed to explore the arena for 10 min. Mice were placed in the middle chamber at the beginning of each phase. Activity was tracked with EthoVision XT 17 software to record distance traveled and time spent in each compartment of the arena during both the habituation and sociability phases, as well as the frequency of direct interactions with the cylinders during the sociability phase. Mice were excluded from the sociability phase data analysis if they failed to stay within the arena during the trial.

Prepulse Inhibition

Mice were restrained in a clear acrylic cylinder (length: 12.7 cm × inner diameter: 3.8 cm) inside a startle response box (L × W × H = 33.0 cm × 33.0 cm × 33.0 cm. SR-LAB, San Diego Instruments, San Diego, CA). An accelerometer attached underneath the tube restraint recorded movements. Mice were habituated to the isolation cabinet for 5 min with a background white noise of 65 dB, which remained consistent throughout the entire experiment. Each mouse underwent 72 trials in total, in which the first and last 6 trials (Block I and Block IV) consisted solely of pulse-

only trials to verify that no habituation to the startle pulse occurred during the experiment. All trials had a randomly spaced inter-trial interval ranging from 8-23 sec. The startle pulse was set to 120 dB, and the prepulse intensities were set to 70 dB (PPI 5), 75 dB (PPI 10), and 80 dB (PPI 15). Each startle tone was 40 msec, and each prepulse was 20 msec. The prepulse preceded the startle by 80 msec, and data were recorded for 65 msec after the startle started. The startle response was recorded in millivolts in SR-LAB software. Percent prepulse inhibition was calculated, normalized to the startle response of the pulse alone trials from Blocks II and III, as follows: percent prepulse inhibition = (startle response for pulse alone − startle response for pulse with prepulse)/startle response for pulse alone × 100.

EEG and EMG Headmount Implantation and Monitoring

Procedures followed published methods (6). Briefly, mice were anesthetized with isoflurane (3% for induction, 1-1.5% for maintenance) and placed in a stereotaxic frame. Eyes were protected with ophthalmic ointment, meloxicam was given (2 mg/kg, s.c.), and body temperature was maintained on a heating pad. After a midline scalp incision, the skull was cleaned and dried. A modular three-channel head-mount (2 EEG/1 EMG) (8201, Pinnacle Technology, Lawrence, KS) was centered on the skull so that the front holes of the headmount were situated between the frontonasal and coronal sutures and the rear holes of the headmount were between the coronal and lambdoid sutures. Four burr holes were drilled and four stainless steel screws were secured; EMG electrodes were inserted bilaterally into the nuchal muscles. The headmount was secured with dental acrylic, the skin was sutured, and meloxicam was continued for 2 days. Mice recovered for 5 days before recording.

For continuous recordings, mice were placed in a circular mouse cage (8288, Pinnacle Technology). A preamplifier and commutator (Pinnacle Technology) were attached to the headmount. The signals were amplified 100 times, band-pass filtered between 0.5-300 Hz for EEG and 10-300 Hz for EMG and digitized at 1000 Hz via a data acquisition/conditioning system (8206, Pinnacle Technology). Analyses were done with Sirenia Seizure Pro software. Spontaneous seizure activity was analyzed in the EEG2 channel using a 20 sec analysis window with a 1 sec step size. The match was set to > 100% and no upper bound limits were applied. Seizures were detected using the line length method, inputting different thresholds until the highest number of seizure events were marked. All identified seizure events were manually verified.

Maximal Electroshock (MES) Seizure Induction

This protocol has been described previously (6). In short, mice were acclimated to both the apparatus and saline-moistened ear-clip electrodes for ≥3 h/day on two consecutive days. On the testing day, current was increased from 0 mA in 1 mA steps every 2 min until a generalized tonic–clonic seizure with hind-limb extension occurred. Outcomes included mortality, seizure-free proportion, current at seizure induction, and extension-to-flexion (E/F) ratio, defined as time in hind-limb extension (>90°) divided by time in flexion (≤90°), scored from video.

References

1. Stessman HAF, Xiong B, Coe BP, Wang T, Hoekzema K, Fenckova M, et al. Targeted sequencing identifies 91 neurodevelopmental-disorder risk genes with autism and developmental-disability biases. Nat Genet. 2017 Apr;49(4):515–26.
2. Pinkert CA. Transgenic Animal Technology: A Laboratory Handbook. 2nd ed. Elsevier; 2002.
3. Miura H, Quadros RM, Gurumurthy CB, Ohtsuka M. Easi-CRISPR for creating knock-in and conditional knockout mouse models using long ssDNA donors. Nat Protoc. 2018 Jan;13(1):195–215.
4. Clarkson RL, Liptak AT, Gee SM, Sohal VS, Bender KJ. D3 Receptors Regulate Excitability in a Unique Class of Prefrontal Pyramidal Cells. J Neurosci. 2017 June 14;37(24):5846–60.
5. Friard O, Gamba M. BORIS : a free, versatile open-source event-logging software for video/audio coding and live observations. Fitzjohn R, editor. Methods Ecol Evol. 2016 Nov;7(11):1325–30.
6. Petrucci AN, Joyal KG, Chou JW, Li R, Vencer KM, Buchanan GF. Post-ictal Generalized EEG Suppression is reduced by Enhancing Dorsal Raphe Serotonergic Neurotransmission. Neuroscience. 2021 Jan;453:206–21.

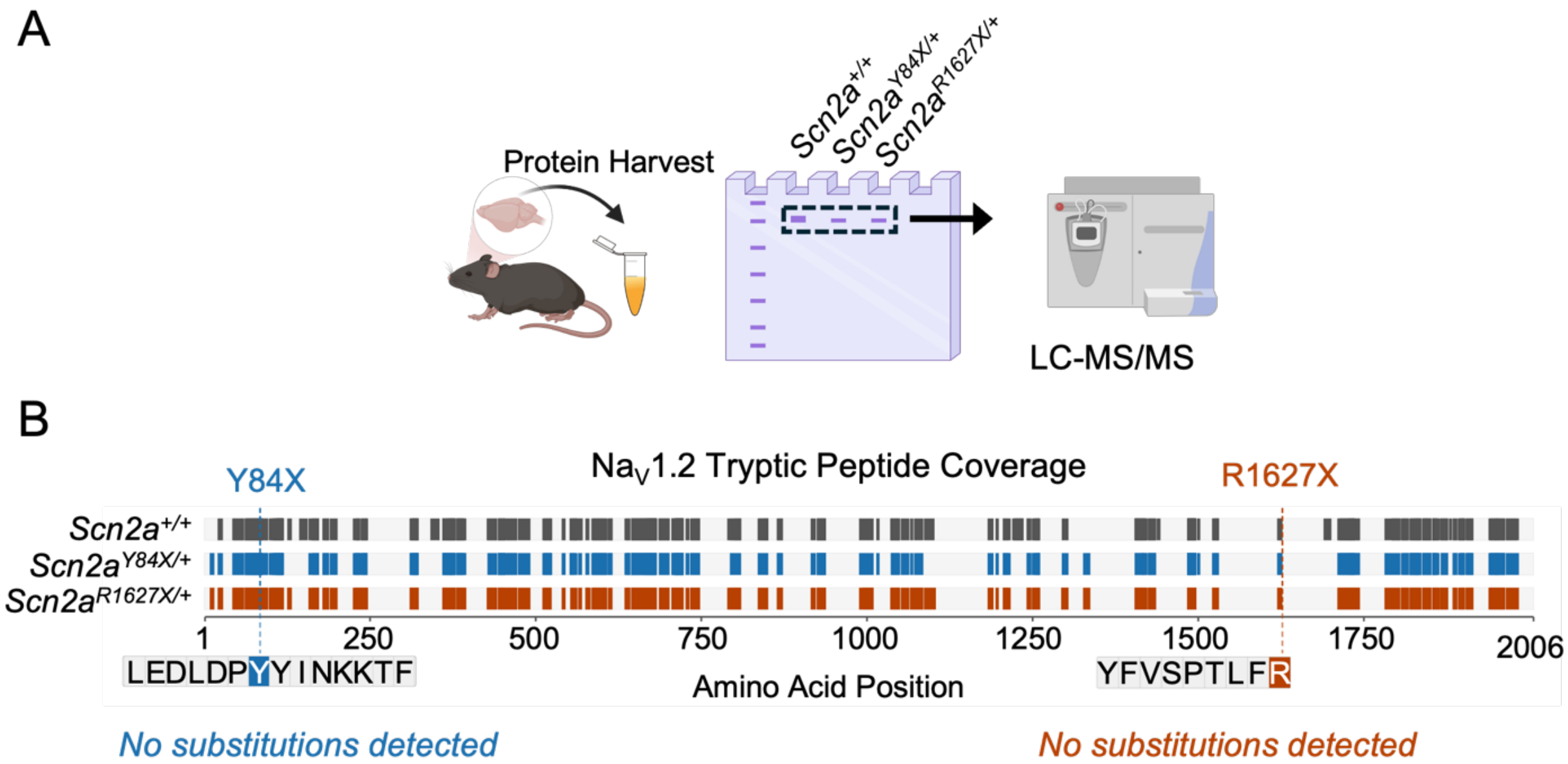

Supplementary Figure 1. Mass spectrometry confirms *Scn2a* sequence fidelity in mouse brains. (A) Gel excision schematic of full-length Na$_V$1.2 from mouse brain tissue. **(B)** LC-MS/MS of full-length Na$_V$1.2 from WT (black), *Scn2a*$^{Y84X/+}$ (blue) and *Scn2a*$^{R1627X/+}$ (red) mouse brain lysate (n = 3 per sample). Gray segments indicate hydrophobic regions lacking peptide coverage. Amino acid sequence flanking each premature termination codon (Tyr84/UAA and Arg1627/UGA) is reconstructed from overlapping tryptic peptides; neither position carries a substitution in any sample, reflecting wild-type allele-derived protein only.

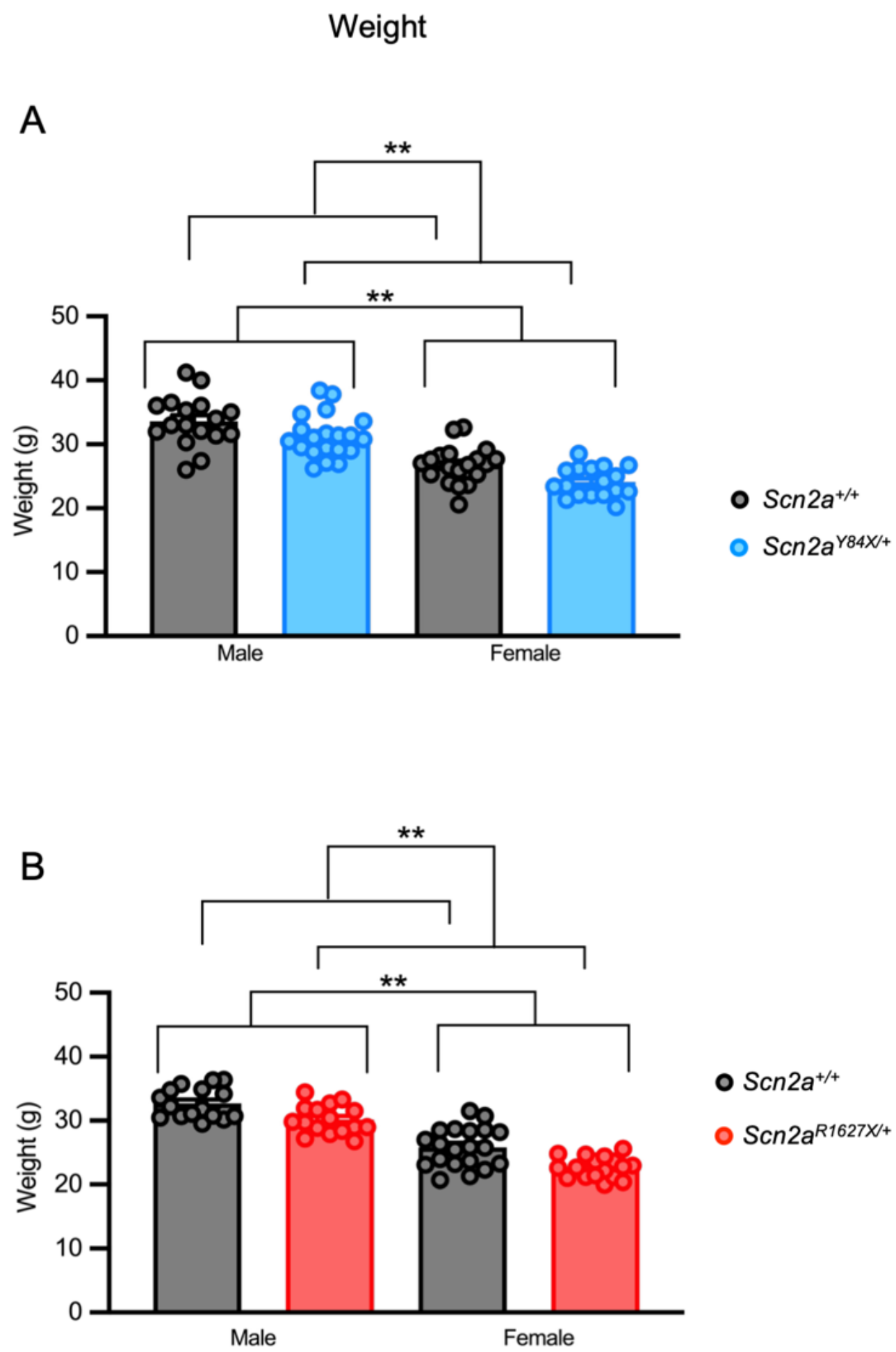

Supplementary Figure 2. *Scn2a*$^{Y84X/+}$ and *Scn2a*$^{R1627X/+}$ mice are on average smaller than their WT littermates. (**A)** In the Y84X line, *Scn2a*$^{Y84X/+}$ mice are on average 2.03 grams smaller than WT mice. Females were on average 6.81 grams smaller than males. **(B)** In the R1627X line, *Scn2a*$^{R1627X/+}$ mice are on average 2.31 grams smaller than WT mice. Females were on average 7.06 grams smaller than males. **(A-B)** Data are expressed as mean ± SEM. **(A)** n = 73 (WTs: n = 17 males, n = 19 females; *Scn2a*$^{Y84X/+}$: n = 20 males, n = 17 females). **(B)** n = 69 (WTs: n = 16 males, n = 20 females; *Scn2a*$^{R1627X/+}$: n = 16 males, n = 17 females).

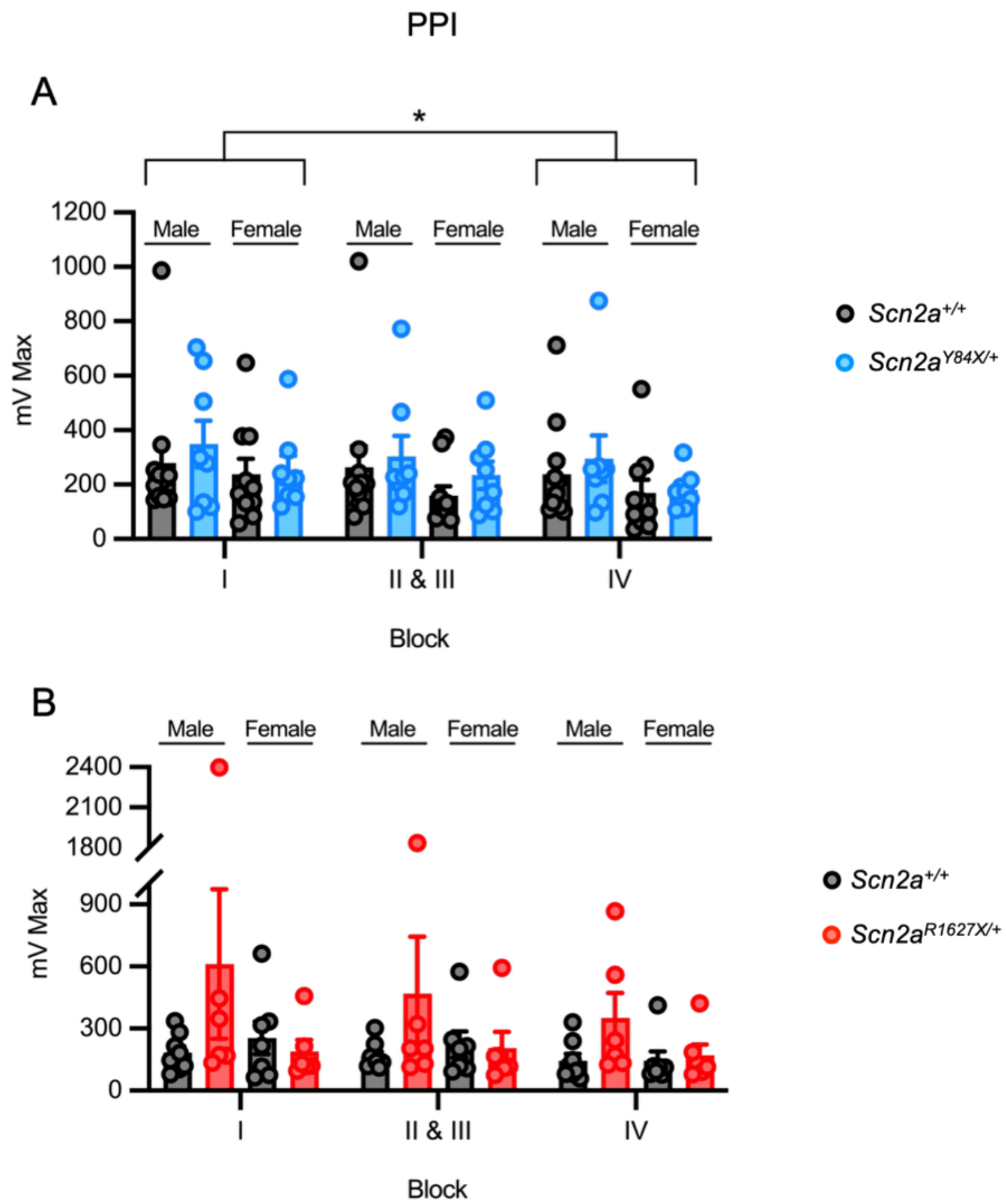

Supplementary Figure 3. There is a mild habituation to the auditory startle in the Y84X line. **(A)** All mice in the Y84X line had a decrease in startle response over the course of the experiment, with no difference between genotypes. **(B)** There were no differences in the auditory startle response in the R1627X line. **(A-B)** Data are expressed as mean ± SEM. **(A)** n = 27 (WTs: n = 8 males, n = 7 females; *Scn2a*$^{R1627X/+}$: n = 6 males, n = 6 females). **(B)** n = 37 (WTs: n = 11 males, n = 10 females; *Scn2a*$^{Y84X/+}$: n = 8 males, n = 8 females).

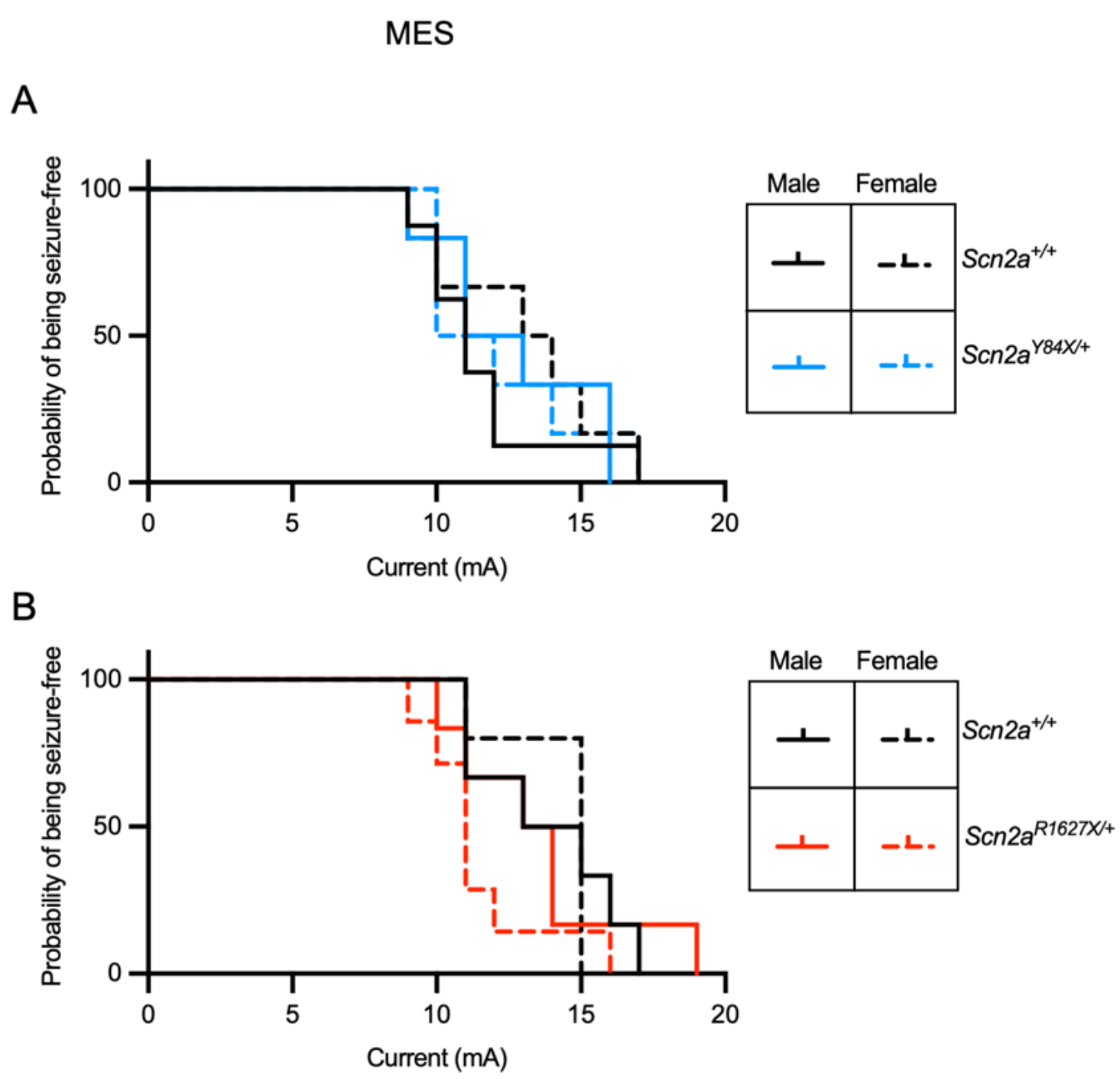

Supplementary Figure 4. There are no differences in the probability of being seizure-free in *Scn2a* PTC mice. (A) There were no differences observed in probability of being seizure-free in the Y84X line or **(B)** the R1627X line. **(A-B)** Data are expressed as mean ± SEM. **(A)** *n* = 26 (WTs: *n* = 8 males, *n* = 6 females; *Scn2a*$^{Y84X/+}$: *n* = 6 males, *n* = 6 females). **(B)** *n* = 24 (WTs: *n* = 6 males, *n* = 5 females; *Scn2a*$^{R1627X/+}$: *n* = 6 males, *n* = 7 females).

Supplementary Table 1. Effects of genotype and sex on weight in Y84X and R1627X mice

Mouse line	Effect	NumDF, DenDF	F-value	*P*-value
**Y84X**	Genotype	1, 69	11.39	**< 0.01**
	Sex	1, 69	87.87	**< 0.01**
	Genotype × Sex	1, 69	0.067	0.796
**R1627X**	Genotype	1, 65	23.83	**< 0.01**
	Sex	1, 65	150.0	**< 0.01**
	Genotype × Sex	1, 65	0.289	0.593

Two-way ANOVA results for effects of genotype, sex, and genotype × sex interaction on body weight in Y84X and R1627X lines. Bold values indicate statistically significant effects ($p < 0.05$).

Supplementary Table 2. Effects of genotype and sex on distance traveled in the elevated zero maze (EZM) and open field test (OFT)

Task	Mouse line	Measure	Effect	NumDF, DenDF	F-value	*P*-value
**EZM**	Y84X	Distance traveled	Genotype	1, 29	1.686	0.204
			Sex	1, 29	0.091	0.765
			Genotype × Sex	1, 29	2.252	0.144
	R1627X		Genotype	1, 37	0.638	0.429
			Sex	1, 37	7.007	**0.012**
			Genotype × Sex	1, 37	0.239	0.628
**OFT**	Y84X	Distance traveled	Genotype	1, 32	0.424	0.519
			Sex	1, 32	0.003	0.958
			Genotype × Sex	1, 32	0.002	0.961
	R1627X		Genotype	1, 38	3.119	0.085
			Sex	1, 38	0.559	0.459
			Genotype × Sex	1, 38	0.038	0.846

Two-way ANOVA results for effects of genotype, sex, and genotype × sex interaction on distance traveled in the EZM and OFT. Bold values indicate statistically significant effects ($p < 0.05$).

Supplementary Table 3. Effects of genotype and sex on anxiety-like behavior in the elevated zero maze (EZM) and open field test (OFT)

Task	Mouse line	Measure	Effect	NumDF, DenDF	F-value	*P*-value
**EZM**	Y84X	Time in open area	Genotype	1, 29	9.541	**< 0.01**
			Sex	1, 29	1.916	0.177
			Genotype × Sex	1, 29	1.132	0.296
	R1627X		Genotype	1, 37	0.175	0.678
			Sex	1, 37	0.563	0.458
			Genotype × Sex	1, 37	0.187	0.668
**OFT**	Y84X	Time in inner area	Genotype	1, 32	0.000	0.987
			Sex	1, 32	1.447	0.238
			Genotype × Sex	1, 32	0.748	0.394
	R1627X		Genotype	1, 38	3.010	0.091
			Sex	1, 38	0.062	0.805
			Genotype × Sex	1, 38	1.545	0.221

Two-way ANOVA and ranked two-way ANOVA results for effects of genotype, sex, and genotype × sex interaction on anxiety-like behavior in the EZM and OFT. Bold values indicate statistically significant effects ($p < 0.05$).

Supplementary Table 4. Effects of genotype and sex in motor learning on the accelerated rotarod task

Mouse line	Effect	NumDF, DenDF	F-value	*P*-value
**Y84X**	Genotype	1, 32	1.47	0.234
	Sex	1, 32	3.609	0.067
	Session	4, 128	54.55	**< 0.01**
	Genotype × Sex	1, 32	1.495	0.230
	Genotype × Session	4, 128	0.636	0.638
	Sex × Session[a]	4, 128	4.189	**< 0.01**
	Genotype × Sex × Session[b]	4, 128	4.639	**< 0.01**
**R1627X**	Genotype	1, 38	2.289	0.139
	Sex	1, 38	0.222	0.640
	Session	4, 152	62.63	**< 0.01**
	Genotype × Sex	1, 38	0.118	0.733
	Genotype × Session	4, 152	1.125	0.347
	Sex × Session	4, 152	0.868	0.485
	Genotype × Sex × Session	4, 152	0.426	0.789

Linear-mixed effects model results for effects of genotype, sex, and interactions in latency to fall on the accelerated rotarod task. Bold values indicate statistically significant effects ($p < 0.05$). Post hoc tests were conducted following significant interactions using estimated marginal means.

[a]Post hoc comparisons were conducted to follow-up the significant sex × session interaction in the Y84X line. We observed a significant difference between males and females on session 5 (session 1, $t_{69.8} = 0.165$, $p = 0.869$, session 2, $t_{69.8} = 1.082$, $p = 0.283$, session 3, $t_{69.8} = 0.830$, $p = 0.409$, session 4, $t_{69.8} = 1.960$, $p = 0.054$, session 5, $t_{69.8} = 3.649$, $p < 0.01$).

[b]Post hoc comparisons were conducted to follow-up the significant genotype × sex × session interaction in the Y84X line. Among females, no significant differences were observed between *Scn2a*$^{Y84X/+}$ and WT mice (session 1, $t_{69.8}$ = 0.561, p = 0.577, session 2, $t_{69.8}$ = 1.848, p = 0.069, session 3, $t_{69.8}$ = -1.068, p = 0.289, session 4, $t_{69.8}$ = -1.158, p = 0.251, session 5, $t_{69.8}$ = -0.214, p = 0.832). In males, there was a significant difference between *Scn2a*$^{Y84X/+}$ and WT mice on sessions 3 and 4, which resolved on session 5 (session 1, $t_{69.8}$ = 1.008, p = 0.317, session 2, $t_{69.8}$ = 0.624, p = 0.535, session 3, $t_{69.8}$ = 2.179, p = 0.033, session 4, $t_{69.8}$ = 2.394, p = 0.019, session 5, $t_{69.8}$ = 0.567, p = 0.573).

Supplementary Table 5. Effects of genotype and sex on grooming behavior

Mouse line	Measure	Effect	NumDF, DenDF	F-value	*P*-value
Y84X	Grooming frequency	Genotype	1, 32	4.018	0.054
		Sex	1, 32	2.081	0.159
		Genotype × Sex	1, 32	0.009	0.926
R1627X		Genotype	1, 38	11.93	**< 0.01**
		Sex	1, 38	0.030	0.864
		Genotype × Sex[a]	1, 38	4.216	**0.047**
Y84X	Time spent grooming	Genotype	1, 32	5.071	**0.031**
		Sex	1, 32	0.702	0.408
		Genotype × Sex	1, 32	1.243	0.273
R1627X		Genotype	1, 38	11.39	**< 0.01**
		Sex	1, 38	0.029	0.865
		Genotype × Sex	1, 38	0.479	0.493

Two-way ANOVA and ranked two-way ANOVA results for effects of genotype, sex, and genotype × sex interaction on grooming behaviors in the OFT. Bold values indicate statistically significant effects ($p < 0.05$). Post hoc tests were conducted following significant interactions using uncorrected Fisher's LSD.

[a]Post hoc comparisons were conducted to follow-up the significant genotype × sex interaction in the R1627X line. Among females, no significant differences were observed between *Scn2a*$^{R1627X/+}$ and WT mice ($t_{38} = 0.998$, $p = 0.325$). In males, there was a significant difference between *Scn2a*$^{R1627X/+}$ and WT mice ($t_{38} = 3.475$, $p < 0.01$).

Supplementary Table 6. Effects of genotype and sex on distance traveled and sociability in the three-chamber social task (3-CST)

(A) Group effects: linear-mixed effects model on distance traveled and sociability

Mouse line	Measure	Effect	NumDF, DenDF	F-value	*P*-value
Y84X	Distance traveled in habituation and sociability phases	Genotype	1, 32.44	0.132	0.718
		Sex	1, 32.44	0.313	0.579
		Phase	1, 30.88	174.278	**< 0.01**
		Genotype × Sex	1, 32.44	1.724	0.198
		Genotype × Phase	1, 30.88	2.009	0.167
		Sex × Phase	1, 30.88	2.682	0.112
		Genotype × Sex × Phase	1, 30.88	0.012	0.915
R1627X		Genotype	1, 23	0.974	0.334
		Sex	1, 23	3.156	0.088
		Phase	1, 23	73.67	**< 0.01**
		Genotype × Sex	1, 23	2.489	0.128
		Genotype × Phase	1, 23	0.095	0.760
		Sex × Phase	1, 23	0.395	0.536
		Genotype × Sex × Phase	1, 23	3.327	0.081
Y84X	Social preference in sociability phase	Genotype	1, 31	0.000	0.999
		Sex	1, 31	0.151	0.701
		Cylinder	1, 31	42.21	**< 0.01**
		Genotype × Sex	1, 31	0.044	0.835

	Genotype × Cylinder	1, 31	0.193	0.664
	Sex × Cylinder	1, 31	0.212	0.649
	Genotype × Sex × Cylinder	1, 31	1.471	0.234
R1627X	Genotype	1, 46	0.761	0.388
	Sex	1, 46	0.438	0.511
	Cylinder	1, 46	65.32	**< 0.01**
	Genotype × Sex	1, 46	0.793	0.378
	Genotype × Cylinder	1, 46	0.030	0.863
	Sex × Cylinder	1, 46	0.016	0.901
	Genotype × Sex × Cylinder	1, 46	0.734	0.396

(B) Social preference: paired t-test within groups

**Mouse line**	**Measure**	**Sex and genotype**	**t(DF)**	**P-value**
Y84X	Social vs object cylinder	Male WT	t(10) = 5.860	**< 0.01**
		Male *Scn2a*$^{Y84X/+}$	t(5) = 3.710	**0.014**
		Female WT	t(9) = 1.865	0.095
		Female *Scn2a*$^{Y84X/+}$	t(8) = 3.60	**< 0.01**
R1627X		Male WT	t(7) = 3.657	**< 0.01**
		Male *Scn2a*$^{R1627X/+}$	t(5) = 5.389	**< 0.01**
		Female WT	t(6) = 5.622	**< 0.01**
		Female *Scn2a*$^{R1627X/+}$	t(5) = 3.120	**0.026**

Linear mixed effects model and linear model used to assess the effects of genotype and sex on distance traveled and time spent with each cylinder in the sociability phase of the 3-

CST. Paired t-test results to compare time within-group preference for the social versus object stimuli. Bold values indicate statistically significant effects (*p* < 0.05).

Supplementary Table 7. Effects of genotype and sex on sensorimotor gating and startle response during the prepulse inhibition task (PPI)

Mouse line	Measure	Effect	NumDF, DenDF	F-value	*P*-value
Y84X	Percent of prepulse inhibition	Genotype	1, 33	1.687	0.203
		Sex	1, 33	2.843	0.101
		Decibel	2, 66	73.88	**< 0.01**
		Genotype × Sex	1, 33	1.00	0.325
		Genotype × Decibel	2, 66	0.296	0.745
		Sex × Decibel	2, 66	0.189	0.828
		Genotype × Sex × Decibel	2, 66	1.421	0.249
R1627X		Genotype	1, 23	0.421	0.523
		Sex	1, 23	0.559	0.461
		Decibel	2, 46	66.49	**< 0.01**
		Genotype × Sex	1, 23	0.835	0.370
		Genotype × Decibel	2, 46	0.029	0.971
		Sex × Decibel	2, 46	0.188	0.829
		Genotype × Sex × Decibel	2, 46	0.416	0.662
Y84X	Startle response	Genotype	1, 33	0.570	0.456
		Sex	1, 33	1.888	0.179
		Block	2, 66	4.453	**0.015**
		Genotype × Sex	1, 33	0.033	0.857
		Genotype × Block	2, 66	0.170	0.844

	Sex × Block	2, 66	0.191	0.827
	Genotype × Sex × Block	2, 66	0.749	0.477
R1627X	Genotype	1, 23	1.515	0.231
	Sex	1, 23	1.069	0.312
	Block	2, 46	3.028	0.058
	Genotype × Sex	1, 23	1.938	0.177
	Genotype × Block	2, 46	0.283	0.755
	Sex × Block	2, 46	0.547	0.583
	Genotype × Sex × Block	2, 46	1.623	0.208

Linear-mixed effects model results for effects of genotype, sex, and interactions in percent of prepulse inhibition and startle response in PPI task. Bold values indicate statistically significant effects ($p < 0.05$).

Supplementary Table 8. Effects of genotype and sex on mortality, current at which seizures were induced, and extension-to-flexion (E/F) ratio in the maximal electroshock (MES) assay

Mouse line	Measure	Effect	Statistic	DF	Effect size/CI	*P*-value
Y84X	Mortality	Genotype	OR = 13.14	–	CI [1.268, 704.98]	**0.014**
		Sex	OR = 3.476	–	CI [0.513, 29.87]	0.217
R1627X		Genotype	χ^2 = 4.086	1	Cramer's V = 0.497	**0.043**
		Sex	χ^2 = 0.671	1	Cramer's V = 0.251	0.413
Y84X	Current at which seizures were induced	Genotype	F = 0.050	1, 22	–	0.825
		Sex	F = 0.166	1, 22	–	0.688
		Genotype × Sex	F = 0.649	1, 22	–	0.429
R1627X		Genotype	F = 3.508	1, 20	–	0.076
		Sex	F = 0.494	1, 20	–	0.490
		Genotype × Sex	F = 0.942	1, 20	–	0.343
Y84X	E/F ratio	Genotype	F = 0.324	1, 20	–	0.575
		Sex	F = 0.029	1, 20	–	0.866
		Genotype × Sex	F = 2.027	1, 20	–	0.170
R1627X		Genotype	F = 2.059	1, 20	–	0.167
		Sex	F = 0.359	1, 20	–	0.555

		Genotype × Sex	F = 0.639	1, 20	–	0.434
Y84X	Chance of being-seizure free	Genotype	χ^2 = 0.000	1	–	0.900
		Sex	χ^2 = 0.300	1	–	0.600
R1627X		Genotype	χ^2 = 1.600	1	–	0.200
		Sex	χ^2 = 0.800	1	–	0.400

Fisher's exact test, Chi-square test of independence, ranked two-way ANOVA, two-way ANOVA, and log-rank test results for effects of genotype, sex, and interactions in mortality, current at which seizures were induced, E/F ratio, and chance of being seizure-free in the MES threshold assay. Bold values indicate statistically significant effects ($p < 0.05$).
